# Post‐Translational Modifications of RNA‐Modifying Proteins in Cellular Dynamics and Disease Progression

**DOI:** 10.1002/advs.202406318

**Published:** 2024-10-08

**Authors:** Yunfan Lin, Pei Lin, Ye Lu, Jiarong Zheng, Yucheng Zheng, Xiangyu Huang, Xinyuan Zhao, Li Cui

**Affiliations:** ^1^ Stomatological Hospital, School of Stomatology Southern Medical University Guangzhou Guangdong 510280 China; ^2^ Department of Dentistry, The First Affiliated Hospital Sun Yat‐Sen University Guangzhou 510080 China; ^3^ School of Dentistry University of California, Los Angeles Los Angeles CA 90095 USA

**Keywords:** post‐translational modifications, RNA‐modifying proteins, therapeutic target

## Abstract

RNA‐modifying proteins, classified as “writers,” “erasers,” and “readers,” dynamically modulate RNA by adding, removing, or interpreting chemical groups, thereby influencing RNA stability, functionality, and interactions. To date, over 170 distinct RNA chemical modifications and more than 100 RNA‐modifying enzymes have been identified, with ongoing research expanding these numbers. Although significant progress has been made in understanding RNA modification, the regulatory mechanisms that govern RNA‐modifying proteins themselves remain insufficiently explored. Post‐translational modifications (PTMs) such as phosphorylation, ubiquitination, and acetylation are crucial in modulating the function and behavior of these proteins. However, the full extent of PTM influence on RNA‐modifying proteins and their role in disease development remains to be fully elucidated. This review addresses these gaps by offering a comprehensive analysis of the roles PTMs play in regulating RNA‐modifying proteins. Mechanistic insights are provided into how these modifications alter biological processes, contribute to cellular function, and drive disease progression. In addition, the current research landscape is examined, highlighting the therapeutic potential of targeting PTMs on RNA‐modifying proteins for precision medicine. By advancing understanding of these regulatory networks, this review seeks to facilitate the development of more effective therapeutic strategies and inspire future research in the critical area of PTMs in RNA‐modifying proteins.

## Graphical abstract

Post‐translational modifications (PTMs) of RNA‐modifying proteins, such as METTL3 and METTL14, modulate their activity and interactions, impacting transcription, signaling pathways, and immune responses. These processes are crucial in driving cancer progression, immune evasion, and pathogen responses, making PTMs of RNA modifiers potential therapeutic targets for clinical intervention in various diseases. This underscores their significance in both cellular dynamics and disease treatment strategies.

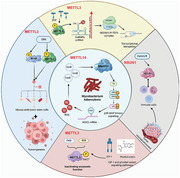

## 1 Introduction

**Figure 1:**
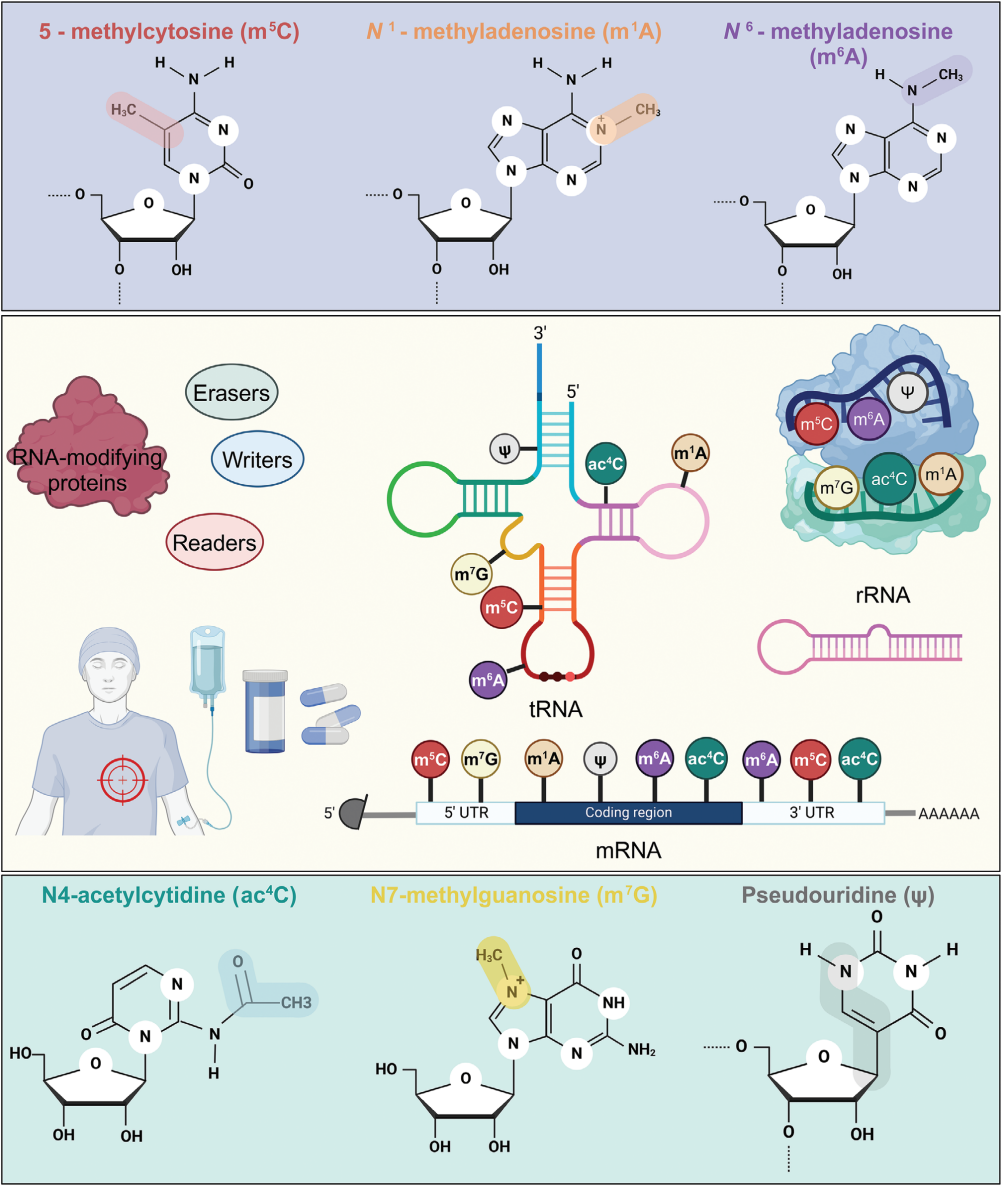
The various chemical modifications found on RNA molecules and their roles in cellular processes. The top panel displays the structures of key RNA modifications: m^5^C, m^1^A, and m^6^A. These modifications occur at specific nucleotides and play crucial roles in the regulation of RNA function. The bottom panel displays the chemical structures of ac^4^C, m^7^G, and Ψ, underscoring the diversity and complexity of RNA chemical modifications. The central section depicts the modification landscape of different types of RNA, including tRNA, rRNA, and mRNA. The modifications are indicated as colored circles on the RNA sequences: m^5^C, m^1^A, m^6^A, ac^4^C, m^7^G, and Ψ. The modification sites on tRNA and rRNA are essential for their structural stability and functional efficiency. In addition, it also highlights the interplay between RNA modifying enzymes categorized into “writers,” “erasers,” and “readers” that respectively add, remove, and recognize these modifications.

RNA modifications act as a sophisticated regulatory mechanism that influences gene expression at the post‐transcriptional level. Key players in this process are RNA‐modifying proteins, which function as “writers,” “erasers,” and “readers”—adding, removing, or interpreting chemical groups on RNA to control its functionality, stability, and interactions with other molecules. Modifications such as N^6^‐methyladenosine (m^6^A), N^1^‐methyladenosine (m^1^A), 5‐methylcytosine (m^5^C), N^4^‐acetylcytosine (ac^4^C), and N^7^‐methylguanosine (m^7^G), along with pseudouridylation (Ψ), impact various RNA types including mRNA, tRNA, rRNA, miRNA, and lncRNA.^[^ ^1^, ^2^ ^]^ Specific RNA‐modifying enzymes, such as METTL3 and METTL14, serve as “writers” that catalyze the addition of m^6^A marks, which are crucial for RNA stability, splicing, and translation. “Erasers” like FTO and ALKBH5 remove these modifications, dynamically regulating RNA fate, while “readers” such as the YTHDF family and YTHDC1 interpret these marks, influencing RNA processing and degradation.^[^ ^3^ ^]^ In addition, RNA‐modifying proteins have emerged as significant targets for drug design and precision medicine, particularly in developing therapeutic strategies for diseases caused by abnormal RNA modifications (**Figure** **1**).^[^ ^4^, ^5^, ^6^ ^]^

Post‐translational modifications (PTMs) such as phosphorylation, ubiquitination, acetylation, and methylation critically modify protein activity, stability, localization, and interactions, thereby playing vital roles in maintaining cellular homeostasis and adapting to physiological changes.^[^ ^7^ ^]^ Acetylation of the endoplasmic reticulum (ER)‐phagy receptor FAM134B by CBP enhances ER‐phagy, while SIRT7‐mediated deacetylation regulates its activity to maintain ER homeostasis, highlighting the sophisticated control by PTMs.^[^ ^8^ ^]^ Similarly, UBC9‐mediated SUMOylation enhances CD4^+^ T cell proliferation by modulating PDPK1 and mTORC1 signaling pathways, crucially affecting glycolytic metabolism.^[^ ^9^ ^]^ Interestingly, erythrocyte transglutaminase 2‐mediated PTMs are crucial for oxygen delivery and adaptation to hypoxia by stabilizing bisphosphoglycerate mutase and activating the Rapoport‐Luebering glycolytic shunt. Importantly, dysregulation of PTMs is implicated in disease progression.^[^ ^10^ ^]^ For instance, LDHA‐mediated histone H3 lysine 18 lactylation at the TPI1 promoter regulates glycolysis and exacerbates osteoarthritis progression, as evidenced by enhanced glycolysis markers in LPS‐induced chondrocytes and reduced cartilage injury upon LDHA knockout in vivo.^[^ ^11^ ^]^ In addition, CCT2 ubiquitination by E3 ubiquitin ligase TRIM21 mitigates its pro‐tumor activities in breast cancer, suggesting a therapeutic potential for targeting this PTM to combat tumor progression and immune evasion.^[^ ^12^ ^]^ Moreover, palmitic acid exacerbates tau protein phosphorylation and acetylation in neurons, mediated by GSK3β and mTOR pathways and associated with Alzheimer's disease‐like changes. This highlights the molecular link between saturated fat intake and cognitive impairment through specific PTMs.^[^ ^13^ ^]^ Notably, PTMs extend to RNA modifying protein and dynamically control their ability to write, erase, and read chemical modifications on RNA and ultimately dictate the fate of RNA molecules within the cell. Understanding the PTMs of RNA modifying proteins is crucial for unraveling the intricate mechanisms of RNA metabolism and function and is vital for identifying novel therapeutic approaches to treat diseases linked to RNA dysregulation.

In this review, we provide a comprehensive and critical evaluation of PTMs of RNA modifying proteins, with a focus on their impact on cellular functions and disease progression. Our analysis is distinguished by several unique aspects. First, this review offers the first systematic summary and analysis of PTMs on RNA modifying proteins, providing a novel perspective on how these modifications influence cellular behaviors and disease mechanisms. In addition, we delve into current research on PTMs closely associated with RNA modifying proteins. Finally, we discuss current challenges and propose innovative therapeutic strategies targeting these PTMs on RNA modifying proteins for disease treatment. This review aims to enhance understanding of these complex modifications and to inspire future research and therapeutic innovation.

## 2 Overview of RNA Epigenetic Modification

RNA epigenetics, also known as epitranscriptomics, investigates reversible chemical modifications to RNA molecules that significantly influence gene expression and cellular function without altering the primary RNA sequence. One significant type of modification within this framework is methylation, which affects tRNA, mRNA, and rRNA. Methylation occurs at various nucleotide bases and the 2′ hydroxyl group of ribose, facilitated by RNA methyltransferases. These enzymes, grouped into four main families—DNMT2, NSUN, FTSJ, and METTL—use S‐adenosylmethionine (SAM) as a universal methyl donor. This targeted methylation influences RNA stability, structure, and interactions with other biomolecules, playing a pivotal role in regulating gene expression and cellular metabolism.^[^ ^14^, ^15^ ^]^ Thus, understanding the diverse proteins involved in RNA modifications deepens our comprehension of the complex regulatory networks that influence cellular functions through epitranscriptomic mechanisms.

### 2.1 m^6^A Modification

**Figure 2:**
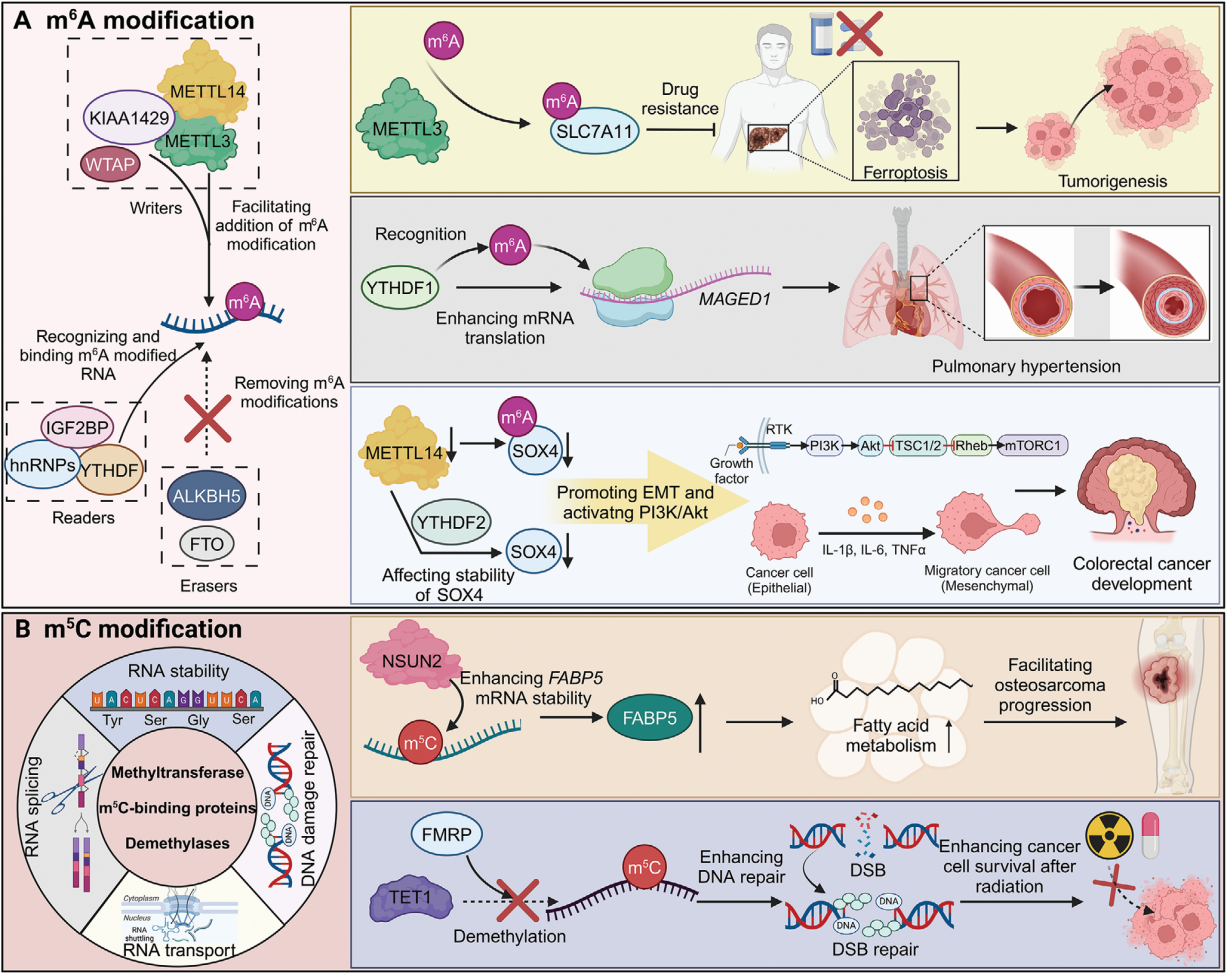
Mechanisms and impacts of m^6^A and m^5^C modifications on RNA and cellular processes. A) The left section outlines the process of m^6^A modification. The “writers” complex, consisting of METTL3, METTL14, WTAP, and KIAA1429, facilitates the addition of m^6^A modifications to RNA. The “readers” such as YTHDF1, IGF2BP, and hnRNPs recognize and bind m^6^A‐modified RNA, influencing its function. “Erasers” like ALKBH5 and FTO remove m^6^A modifications, thus reversing their effects. The top right panel illustrates m^6^A's role in drug resistance and tumorigenesis through the modulation of *SLC7A11*, promoting ferroptosis. The middle right panel shows how m^6^A, recognized by YTHDF1, enhances the translation of *MAGED1*, contributing to pulmonary hypertension. The bottom right panel depicts the stabilization of *SOX4* mRNA by METTL14‐mediated m^6^A modification, promoting EMT and PI3K/Akt pathway activation, leading to colorectal cancer development. B) The left section describes the m^5^C modification mechanism. Methyltransferases such as NSUN2 add the m^5^C mark, which is recognized by m^5^C‐binding proteins and influences RNA stability, DNA damage repair, and transport. Demethylases like TET1 remove these modifications. The top right panel illustrates m^5^C's role in enhancing the stability of *FABP5* mRNA, which is linked to fatty acid metabolism and osteosarcoma progression. The bottom right panel demonstrates the involvement of m^5^C in DNA repair, where demethylation by TET1 facilitates cancer cell survival after radiation therapy by enhancing DSB repair.

m^6^A is a prevalent RNA methylation modification in eukaryotic RNA, crucial for regulating its stability, splicing, localization, and translation (**Figure** **2**A).^[^ ^16^ ^]^ METTL3, the catalytic core, and METTL14, which forms a stable complex with METTL3 to enhance activity, along with WTAP and KIAA1429, facilitate the addition of the m^6^A modification.^[^ ^17^, ^18^, ^19^ ^]^ For instance, METTL3‐mediated m^6^A modification of *SLC7A11* induces resistance to ferroptosis in hepatocellular carcinoma, promoting tumorigenesis.^[^ ^20^ ^]^ In addition, reduced expression of METTL14 decreases the m^6^A modification levels of *SOX4*, and through the “reader” protein YTHDF2, affects the stability of *SOX4*. This ultimately promotes SOX4‐mediated epithelial–mesenchymal transition and activates the SOX4‐driven PI3K/Akt signaling pathway, enhancing colorectal cancer development.^[^ ^21^ ^]^ Erasers like FTO and ALKBH5 remove these modifications, thereby reversing the effects of writers and influencing RNA processes.^[^ ^22^ ^]^

Notably, m^6^A readers can be categorized into nuclear and cytoplasmic readers, each playing pivotal roles within the cell. YTHDC1, a key nuclear member of the highly conserved YTH family of proteins, primarily functions as a nuclear reader of m^6^A‐modified RNA. This protein is strategically localized within the nucleus, where it forms nuclear foci known as YT bodies. These YT bodies, found at transcriptionally active sites adjacent to RNA processing speckles, facilitate the interaction of YTHDC1 with emerging m^6^A‐modified RNA transcripts. YTHDC1 features a YTH domain flanked by intrinsically disordered regions rich in arginine, proline, aspartate, and glutamate. These regions enhance its ability to undergo liquid–liquid phase separation, leading to the formation of biomolecular condensates, which are critical for its function.^[^ ^23^ ^]^ The m^6^A‐dependent functions of YTHDC1 have expanded beyond alternative splicing to include other crucial nuclear processes such as mRNA export, secondary structure switching, alternative polyadenylation, pri‐miRNA processing, mRNA stability regulation, and XIST‐dependent X chromosome inactivation.^[^ ^24^, ^25^ ^]^ For instance, YTHDC1 regulates mRNA splicing and nuclear export, essential for skeletal muscle stem cell activation and proliferation.^[^ ^26^ ^]^ Interestingly, YTHDC1 regulates RNA structure by recognizing and binding to the unfolded stem region of the m^6^A‐modified AUCG hairpin in XIST. This interaction modulates RNA conformation, highlighting YTHDC1's role in controlling lncRNA structure and function.^[^ ^27^ ^]^ In addition, YTHDC1 is essential for XIST‐mediated gene silencing, recognizing m^6^A‐modified residues on XIST.^[^ ^28^ ^]^

The cytoplasmic m^6^A readers, such as YTHDF1, YTHDF2, YTHDF3, YTHDC2, and the IGF2BP family (IGF2BP1, IGF2BP2, and IGF2BP3), orchestrate a sophisticated regulatory network that modulates post‐transcriptional RNA dynamics. YTHDF1, YTHDF2, and YTHDF3, which selectively recognize m^6^A‐modified mRNAs, differentially influence mRNA fate in the cytoplasm.^[^ ^23^, ^29^ ^]^ YTHDF1 promotes the translation of m^6^A‐modified mRNAs by facilitating the assembly of translation initiation complexes, thereby enhancing protein synthesis. YTHDF2, on the other hand, directs m^6^A‐tagged transcripts to decay pathways, accelerating mRNA turnover and thus modulating gene expression levels. YTHDF3 serves a dual role, augmenting the functions of both YTHDF1 and YTHDF2 by modulating translation and decay processes based on cellular conditions. YTHDC2, which combines m^6^A‐binding capability with RNA helicase activity, plays a pivotal role in adjusting the translational output of specific mRNAs. It not only enhances ribosome loading but also facilitates the efficient use of mRNAs for protein synthesis. Meanwhile, the IGF2BP proteins stabilize m^6^A‐modified mRNAs, shielding them from degradation mechanisms that are typically facilitated by YTHDF2.^[^ ^30^ ^]^ These cytoplasmic m^6^A readers localize to RNA granules, influencing key cellular processes and disease development.^[^ ^31^ ^]^ For example, YTHDF1 enhances the translation of *MAGED1* mRNA by recognizing m6A modifications, a mechanism that promotes the proliferation of pulmonary arterial smooth muscle cells and contributes to the pathological changes observed in pulmonary hypertension.^[^ ^32^ ^]^

### 2.2 m^5^C Modification

The m^5^C methyltransferases utilize SAM as a methyl donor to transfer methyl groups to cytosine, forming m^5^C (Figure 2B). To date, more than ten types of RNA m^5^C methyltransferases have been identified, including DNMT2, members of the TRDMT family, and the NSUN family. Among them, the NSUN methyltransferases include several members: NSUN1 to NSUN7 and NSUN5a/b/c. NSUN2 and DNMT2 are the primary writers of mRNA m^5^C modification in animal cells.^[^ ^33^, ^34^ ^]^ For instance, NSUN2 enhances the stability of *FABP5* mRNA via m^5^C modification, upregulating FABP5 expression and thus promoting fatty acid metabolism in osteosarcoma cells, ultimately facilitating the progression of osteosarcoma.^[^ ^35^ ^]^ TET1‐3 proteins have been identified as potential RNA demethylases, with FMRP facilitating TET1‐mediated demethylation of m^5^C in RNA at DNA double‐strand breaks, thereby enhancing repair and cancer cell survival following radiation.^[^ ^36^ ^]^ In addition, mitochondrial DNA and RNA dioxygenase ALKBH1 has also been implicated in the demethylation of cytoplasmic tRNAs.^[^ ^34^ ^]^ In the context of readers, YBX1 has been identified as a cytoplasmic m^5^C reading protein in human cells. YBX1 specifically targets several oncogenic genes containing m^5^C, enhancing the stability of these genes and promoting cancer progression.^[^ ^37^ ^]^

### 2.3 m^1^A Modification

**Figure 3:**
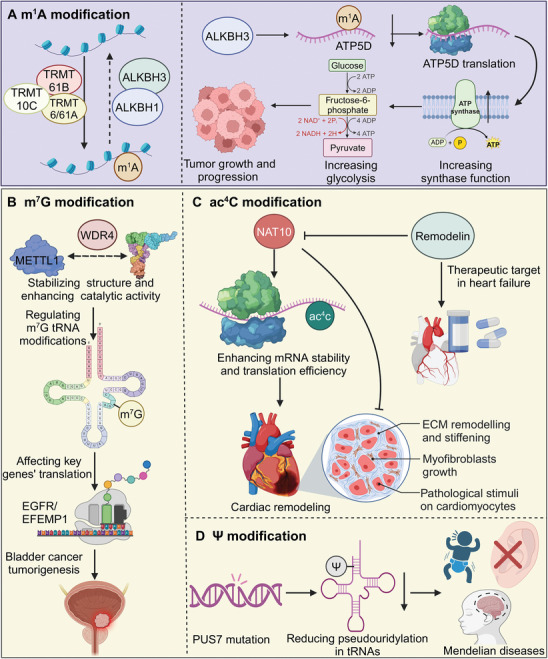
Mechanisms and functional impacts of m^1^A, m^7^G, ac^4^C and Ψ modifications on RNA and cellular processes. A) The METTL1‐WDR4 complex facilitates m^7^G modification, stabilizing RNA structure and enhancing catalytic activity. This modification affects key gene translations such as *EGFR* and *EFEMP1*, and its dysregulation is linked to bladder cancer tumorigenesis. B) The ac^4^C modification, catalyzed by NAT10, enhances mRNA stability and translation efficiency. This process is crucial in cardiac remodeling, where it impacts ECM remodeling, myofibroblast growth, and pathological stimuli on cardiomyocytes. Remodelin, a NAT10 inhibitor, shows promise in treating heart failure by modulating these pathways. C) The impact of Ψ modification on tRNAs is illustrated. Mutations in PUS7 result in reduced pseudouridylation, leading to defective tRNA function and associated Mendelian diseases, characterized by developmental abnormalities. D) The m^1^A modification, regulated by TRMT10C, TRMT61B, and TRMT6/61A, is removed by demethylases ALKBH1 and ALKBH3. In cancer cells, ALKBH3‐mediated m^1^A demethylation of *ATP5D* mRNA promotes its translation, enhancing ATP synthase function and glycolysis, thus supporting tumor growth and progression.

m^1^A is achieved by the methylation of adenine at the N^1^ position (**Figure** **3**A). As a dynamic and reversible RNA modification, m^1^A can be catalyzed by methyltransferases such as TRMT10C, TRMT61B, and TRMT6/61A. Demethylases like ALKBH3 and ALKBH1 act as “erasers,” removing m^1^A modifications.^[^ ^38^ ^]^ For instance, ALKBH3 regulates cancer cell glycolysis by demethylating m^1^A at ATP5D, which is crucial for adenosine 5′‐triphosphate synthase function. This modification affects ATP5D translation and stability, influencing tumor growth and cancer progression.^[^ ^39^ ^]^ Interestingly, the binding of ALKBH3 to m^1^A induces significant conformational changes. The extension of ALKBH3's α2 helix into a β‐hairpin structure stabilizes the m^1^A base substrate in the active pocket, while the β4‐β5 hairpin shifts outward to accommodate single‐stranded nucleic acid substrates and prevent double‐stranded nucleic acid binding.^[^ ^40^ ^]^ Proteins containing the YTH domain, such as YTHDF1‐3 and YTHDC1, directly recognize m^1^A modified RNA. YTHDF2 recognizes m^1^A modified mRNA sequences with specific affinity, influencing the abundance of m^1^A transcripts in cells. Depleting YTHDF2 increases these transcripts, while reducing the ALKBH3 destabilizes them, highlighting YTHDF2's regulatory role in m^1^A‐mediated mRNA stability.^[^ ^41^ ^]^

### 2.4 Other Types of RNA Modification

In addition to above mentioned RNA modifications, ac^4^C, m^7^G, and Ψ are key chemical alterations. m^7^G is a critical RNA modification predominantly found at the 5′ cap structure of eukaryotic mRNAs, where it plays a pivotal role in modulating various aspects of RNA metabolism (Figure 3B). This cap, consisting of an m^7^G moiety linked via a 5′‐5′ triphosphate bridge to the first nucleotide of the transcript, serves as a crucial determinant for mRNA stability, efficient splicing, export from the nucleus, protection from exonucleolytic degradation and translation initiation. Beyond mRNAs, m^7^G modifications are also found in other RNA species such as tRNAs and rRNA. Importantly, m^7^G modification is closely linked to normal cellular homeostasis and the progression of various diseases.^[^ ^42^ ^]^ For instance, tRNA m^7^G46 methylation, catalyzed by METTL1‐WDR4, is critical for maintaining cellular homeostasis by preventing ribosome stalling and ensuring efficient mRNA translation. Loss of METTL1 and m^7^G modifications during senescence triggers rapid tRNA degradation, disrupts translation of key pathways, and activates stress responses that drive senescence phenotypes.^[^ ^43^ ^]^ METTL1, upregulated in failing human and hypertrophic murine hearts, modulates cardiac hypertrophy via m^7^G modification of *SRSF9* mRNA, enhancing SRSF9 expression and NFATc4 splicing. This regulatory mechanism underscores m^7^G's role in heart failure progression and highlights METTL1 as a potential therapeutic target.^[^ ^44^ ^]^ In addition, Upregulation of METTL1 and WDR4 in esophageal squamous cell carcinoma (ESCC) enhances tRNA m^7^G modification, driving cancer progression by boosting translation of oncogenic mRNAs in the RPTOR/ULK1/autophagy pathway. Knockdown of METTL1 or WDR4 reduces m^7^G‐modified tRNAs and suppresses tumor growth, making m^7^G a key driver and potential therapeutic target in ESCC.^[^ ^45^ ^]^

ac^4^C is a conserved RNA modification found on cytidine residues in both tRNA and mRNA, playing a significant role in regulating RNA stability, translation, and overall cellular homeostasis (Figure 3C). ac^4^C is introduced by the enzyme complex composed of NAT10, which acetylates cytidine at N^4^ position.^[^ ^46^ ^]^ In tRNA, ac^4^C enhances codon recognition and fidelity during translation, while in mRNA, it promotes efficient translation and stability by protecting transcripts from degradation. The precise regulation of ac^4^C modification is crucial for maintaining proper cellular function, and its dysregulation has been linked to various pathological conditions.^[^ ^47^ ^]^ For instance, ac^4^C dynamically regulates mRNA during spermatogenesis, with its disruption impairing meiotic entry, chromosome synapsis, and DNA repair in germ cells. Loss of ac^4^C leads to significant defects in male reproduction, highlighting its crucial role in cellular function during meiosis.^[^ ^48^ ^]^ Notably, NAT10‐mediated ac^4^C acetylation enhances mRNA stability and translation, particularly of *CD47* and *ROCK2*, driving cardiac remodeling. Inhibition of NAT10 with Remodelin reduces cardiac hypertrophy, fibrosis, and inflammation, making it a promising therapeutic target in heart failure.^[^ ^49^ ^]^ Similarly, ac^4^C modification by NAT10 enhances hypoxia tolerance in gastric cancer through glycolysis addiction. HIF‐1α regulates NAT10, which modifies *SEPT9* mRNA, driving a positive feedback loop that activates the HIF‐1α pathway.^[^ ^50^ ^]^

Pseudouridine modification involves the conversion of the N‐glycosidic bond in uridine to a C‐glycosidic bond, forming pseudouridine. This transformation significantly enhances the structural stability and functionality of RNA, especially in tRNA and rRNA, and may also impact mRNA function. The formation of pseudouridine is catalyzed by a group of enzymes known as Pseudouridine synthases, including PUS1, PUS4, and PUS7 (Figure 3D).^[^ ^51^ ^]^ Notably, mutations in PUS7 are associated with intellectual disability, progressive microcephaly, and symptoms such as short stature and hearing loss. These mutations lead to reduced pseudouridylation in tRNAs, highlighting PUS7's crucial role in tRNA modification and its link to specific Mendelian diseases.^[^ ^52^ ^]^

Importantly, understanding the similarities and differences between various RNA modifications is crucial for comprehending their distinct roles in cellular processes and their implications in diseases. For instance, m^6^A and m^5^C are pivotal RNA modifications that both contribute to the regulation of RNA stability, translation, and gene expression. These modifications are both dynamically regulated by specific RNA modification enzymes and are implicated in various diseases, particularly cancer. Despite these commonalities, they differ significantly in their chemical structure, prevalence, and regulatory mechanisms. Chemically, m^6^A involves methylation at the nitrogen atom on the 6th position of adenosine, whereas m^5^C involves methylation at the 5th carbon of cytosine. m^6^A is the most prevalent internal modification in mRNA, whereas m^5^C, though less abundant, is distributed across various RNA species, including tRNA, rRNA, mRNA, and non‐coding RNAs. The distinct sets of enzymes that regulate m^6^A and m^5^C further underscore their different roles in RNA biology. These differences highlight the unique yet complementary roles of m^6^A and m^5^C within the broader context of RNA modification.

## 3 Common Types of Post‐Translational Modifications

PTMs are a crucial mechanism for regulating protein function in cells, dynamically altering the chemical properties and biological activities of proteins through the formation or cleavage of covalent bonds on protein backbones or amino acid side chains. These modifications include the addition of small chemical entities such as phosphate, sugar, methyl, and acetyl groups, as well as changes in chemical properties like deamination, deamidation, citrullination, and oxidation. PTMs are crucial in a broad spectrum of biological processes, and abnormalities in PTMs can lead to protein dysfunction, contributing to the onset and progression of diseases such as cancer, neurodegenerative disorders, and metabolic diseases.

### 3.1 Protein Methylation

**Figure 4:**
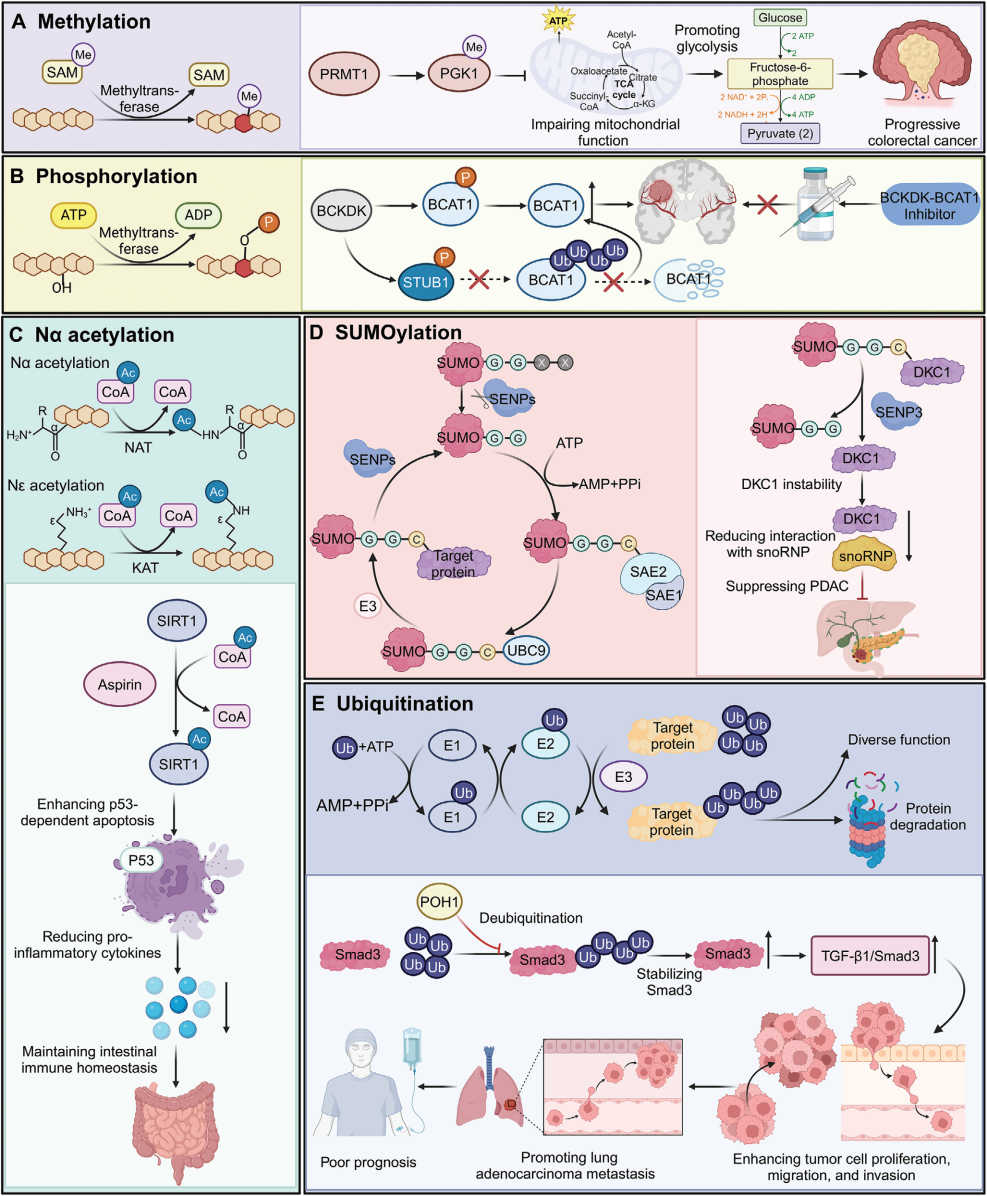
Mechanisms and functional impacts of various post‐translational modifications on proteins and cellular processes. A) The process of protein methylation is depicted, where methyltransferases use SAM to add methyl groups to target proteins. PRMT1‐mediated methylation of PGK1 promotes glycolysis, which impairs mitochondrial function and contributes to the progression of colorectal cancer. B) The phosphorylation process involves kinase‐mediated transfer of phosphate groups from ATP to target proteins. Phosphorylation of BCAT1 by BCKDK regulates its activity. The use of BCKDK‐BCAT1 inhibitors can impede this pathway, offering potential therapeutic interventions. C) Acetylation modifications occur on lysine residues (Nα and Nε acetylation). Nα‐acetylation, catalyzed by NAT, and Nε‐acetylation, catalyzed by KAT, play key roles in protein function. SIRT1 deacetylase, modulated by aspirin, enhances p53‐dependent apoptosis and reduces pro‐inflammatory cytokines, maintaining intestinal immune homeostasis. D) The SUMOylation cycle involves the conjugation of SUMO proteins to target proteins, mediated by E1, E2, and E3 enzymes, and reversed by SENPs. SUMOylation affects DKC1 stability and its interaction with snoRNP, suppressing PDAC. E) The ubiquitination process involves E1, E2, and E3 enzymes tagging target proteins with ubiquitin, marking them for degradation by the proteasome. POH1 deubiquitinase removes ubiquitin from Smad3, stabilizing it and promoting lung adenocarcinoma metastasis by enhancing TGF‐β1/Smad3 signaling, which drives tumor cell proliferation, migration, and invasion.

Protein involves the enzymatic transfer of methyl groups from SAM to specific amino acids such as lysine, arginine, histidine, asparagine, and glutamine. The specificity of protein methylation is determined by the sequence context around the target site and the tertiary structure of the protein, which influences enzyme access and activity. This modification alters protein function by impacting interaction domains, enzyme activity, or protein stability, thereby modulating diverse biological processes.^[^ ^53^ ^]^ Protein methylation critically influences interactions with DNA, RNA, or other proteins by increasing hydrophobicity without changing the amino acid's charge. Specifically, arginine methylation, catalyzed by protein arginine methyltransferases (PRMTs) targeting GAR motifs, can be reversed by Jumonji C‐terminal domain family demethylases, including KDM3A, KDM4E, and KDM5C.^[^ ^54^ ^]^ Similarly, lysine methylation occurs in histones and some non‐histone proteins and is catalyzed by lysine methyltransferases such as SET domain‐containing enzymes, it is reversed by lysine‐specific demethylases like LSD1 or other JmjC domain‐containing proteins. The specific sites and degrees of methylation on histone tails dictate distinct transcriptional outcomes. Abnormal protein methylation has been associated with various diseases.^[^ ^55^ ^]^ For instance, PRMT1‐mediated methylation at arginine 206 on PGK1 enhances phosphorylation at serine 203, impairing mitochondrial function and promoting glycolysis in colorectal cancer cells.^[^ ^56^ ^]^ Similarly, CFP1 regulates cardiomyocyte maturation through histone H3K4me3 modification, impacting gene expression related to structural, metabolic, and contractile functions (**Figure** **4**A).^[^ ^57^ ^]^

### 3.2 Protein Phosphorylation

Protein phosphorylation is mediated by kinases, which are enzymes that specifically target sequence motifs containing serine, threonine, or tyrosine residues. These kinases recognize sequences where specific amino acids enhance binding affinity and substrate specificity. Upon binding, the kinase transfers a phosphate group from ATP to the target amino acid's hydroxyl group, forming a covalent phosphoester bond.^[^ ^58^ ^]^

Phosphorylation introduces a negatively charged phosphate group into a protein, significantly altering its properties. This addition can induce conformational changes that either expose or obscure interaction domains and active sites, thereby modulating the protein's function—activating or inhibiting it based on the context. For example, in the MAP kinase cascade, phosphorylation activates a series of kinases in sequence, amplifying the signal. Phosphorylation also creates docking sites for proteins that recognize phosphorylated motifs, leading to the assembly of complexes that drive crucial cellular processes. The reversible nature of phosphorylation, controlled by phosphatases that remove phosphate groups, allows for dynamic regulation. Disruptions in phosphorylation dynamics, such as in cancer, involve aberrant kinase or phosphatase activities, leading to uncontrolled cell proliferation or impaired apoptosis.^[^ ^59^, ^60^ ^]^ For instance, phosphorylation by BCKDK enhances BCAT1's catalytic and antioxidant activities and stability in glioblastoma (GBM), and also phosphorylates the E3 ubiquitin ligase STUB1 to prevent BCAT1 degradation by disrupting their interaction. This mechanism promotes GBM proliferation and increases sensitivity to chemotherapy with temozolomide when inhibited (Figure 4B).^[^ ^61^ ^]^

### 3.3 Protein Acetylation

Protein acetylation involves the addition of acetyl groups to lysine residues and the N‐terminus of proteins, mediated by a family of enzymes known as N‐terminal acetyltransferases (NATs). Each NAT, from NatA through NatG, recognizes distinct sequences at the N‐terminus, largely dictated by the initial few amino acids. The irreversible nature of this modification significantly alters the protein's interaction landscape by preventing N‐terminal degradation and modulating binding affinity with other cellular components, thus influencing protein half‐life, cellular localization, and involvement in protein complexes.^[^ ^62^ ^]^

Lysine acetylation, in contrast, is reversible and dynamically regulated, primarily controlled by lysine acetyltransferases (KATs) such as the GNAT, MYST, and CBP/p300 families, and lysine deacetylases (KDACs), including the sirtuin family and HDACs. KATs add acetyl groups to the ε‐amino group of lysine residues, reducing the overall positive charge of the protein. This charge reduction affects the protein's interactions with negatively charged DNA or other proteins, influencing DNA accessibility and gene transcription.^[^ ^63^ ^]^ Conversely, deacetylation by KDACs removes these acetyl groups, often restoring the positive charge and promoting tighter DNA binding and transcriptional repression. This dynamic interplay between acetylation and deacetylation is crucial in cellular signaling and regulatory processes, enabling efficient cellular responses to internal and external stimuli.^[^ ^64^ ^]^ This enhances p53‐dependent apoptosis and reduces pro‐inflammatory cytokines, maintaining intestinal immune homeostasis and highlighting therapeutic potential for controlling inflammation (Figure 4C).^[^ ^65^ ^]^

### 3.4 Protein SUMOylation

SUMOylation represents a crucial PTM in eukaryotes, where small ubiquitin‐like modifier (SUMO) proteins are covalently attached to target proteins, profoundly impacting their function and localization. The SUMOylation process begins with the maturation of SUMO precursors, where their C‐terminal amino acids are cleaved by SUMO‐specific proteases (SENPs), exposing a diglycine residue. This mature SUMO is then activated in an ATP‐dependent manner by the E1 activating enzyme complex composed of SAE1 and SAE2. This activation facilitates the transfer of SUMO to the E2 conjugating enzyme UBC9, which plays a central role in recognizing target proteins for SUMOylation, often in conjunction with various E3 ligating enzymes that enhance the efficiency and specificity of the reaction. The dynamic nature of SUMOylation is underscored by its reversibility, conjugated SUMOs can be removed by SENPs, allowing both the SUMO and the substrate protein to participate in subsequent cycles of modification.^[^ ^66^ ^]^ Dysregulation of SUMOylation is linked to various diseases, highlighting its importance in cellular processes and its potential as a therapeutic target.^[^ ^67^, ^68^ ^]^ For example, SENP3 acts as a suppressor in pancreatic ductal adenocarcinoma (PDAC) by deSUMOylating DKC1, leading to its instability and reduced interaction with snoRNP proteins, thus impairing PDAC cell migration (Figure 4D).^[^ ^69^ ^]^

### 3.5 Protein Ubiquitination

Protein ubiquitination is orchestrated by a highly regulated enzymatic cascade that attaches ubiquitin to substrate proteins, significantly impacting their function and cellular fate. This process begins with the ubiquitin‐activating enzyme (E1), which catalyzes the ATP‐dependent activation of ubiquitin, forming a high‐energy thioester bond. The activated ubiquitin is then transferred to an ubiquitin‐conjugating enzyme (E2), setting the stage for the action of the ubiquitin ligase (E3), which confers specificity to the ubiquitination process by targeting proteins for modification based on their recognition motifs or structural features.^[^ ^70^ ^]^

Ubiquitination can be mono‐ubiquitination or poly‐ubiquitination, where chains of ubiquitin are linked through different lysine residues on the ubiquitin itself. K48‐linked polyubiquitination, the most well‐known type, primarily signals for proteasomal degradation, directing tagged proteins to the 26S proteasome where they are recognized, unfolded, and degraded into peptides. Conversely, K63‐linked polyubiquitination typically supports non‐degradative roles such as the assembly of signaling complexes and endosomal sorting and trafficking. Linear (M1) ubiquitination, formed through the head‐to‐tail attachment of ubiquitin molecules, plays a specialized role in inflammatory and immune signaling. The reversibility of ubiquitination is maintained by deubiquitinating enzymes, which remove ubiquitin from proteins.^[^ ^71^ ^]^ Disruptions in ubiquitination pathways are closely linked to diseases such as cancer, neurodegenerative disorders, and immune dysfunctions.^[^ ^72^ ^]^ For instance, POH1, identified as a novel deubiquitinase for Smad3, enhances TGF‐β1/Smad3 signaling and promotes metastasis in lung adenocarcinoma by stabilizing Smad3 (Figure 4E).^[^ ^73^ ^]^

### 3.6 Other Types of PTMs

In addition to above mentioned PTMs, lactylation, glycosylation and S‐nitrosylation are also significant types of PTM. Lactylation is a PTM where lactyl groups derived from lactyl‐CoA are covalently attached to lysine residues within both histone and non‐histone proteins. This modification serves as a critical link between cellular metabolic states—specifically, elevated lactate production during glycolysis—and regulatory mechanisms governing protein function and gene expression. Lactylation at specific sites such as H3K18 on histones leads to chromatin remodeling, enhancing the accessibility of transcription factors and the transcriptional machinery to DNA, thereby promoting gene expression that is responsive to changes in metabolic conditions.^[^ ^74^ ^]^ The impact of lactylation extends beyond chromatin dynamics. In non‐histone proteins, lactylation modifies various functional aspects including protein stability, activity, and localization across different cellular compartments.^[^ ^75^, ^76^ ^]^

Protein glycosylation involves the covalent attachment of carbohydrate moieties to specific amino acid residues on proteins. This complex biochemical process predominantly occurs in the ER and Golgi apparatus, where specialized enzyme systems catalyze the transfer of sugars from donor molecules to acceptor protein sites. Glycosylation can occur at asparagine residues (N‐linked glycosylation) or at serine/threonine residues (O‐linked glycosylation), with each type following distinct biosynthetic pathways. N‐linked glycosylation begins in the ER with the addition of a pre‐assembled oligosaccharide precursor to the asparagine residue within the consensus sequence Asn‐X‐Ser/Thr. This process is mediated by the oligosaccharyltransferase complex, which transfers a 14‐sugar glycan from a lipid‐linked precursor to the nascent protein. The glycan is then modified as it moves through the ER and Golgi, with specific glycosidases and glycosyltransferases trimming and adding sugars to produce a diverse array of glycan structures. O‐linked glycosylation does not involve a consensus sequence and typically starts in the Golgi apparatus. This modification involves the addition of a single N‐acetylgalactosamine (GalNAc) to the hydroxyl group of serine or threonine, catalyzed by a family of enzymes known as polypeptide N‐acetylgalactosaminyltransferases. Subsequent modifications can add more sugars, creating complex O‐glycan structures.^[^ ^77^, ^78^ ^]^ Glycosylation affects protein folding, stability, and trafficking, critical for membrane and secreted proteins. It also influences cell signaling, immune recognition, and adhesion by modulating interactions with biomolecules. Disruptions in glycosylation are linked to diseases like congenital disorders, autoimmune diseases, and cancer.^[^ ^78^ ^]^

Protein S‐nitrosylation is characterized by the covalent attachment of a nitric oxide (NO) group to the thiol side chain of cysteine residues within proteins. This modification occurs through a redox‐mediated reaction that typically involves the nitrosylating agents derived from NO synthesis in cells. S‐nitrosylation is catalyzed by several mechanisms, including direct transfer from nitric oxide itself, trans‐nitrosylation where NO groups are transferred between cysteine residues, and through metalloprotein‐mediated catalysis involving transition metals that facilitate NO group transfer. Biochemically, S‐nitrosylation is reversible and plays a dynamic role in modulating protein function by regulating enzyme activity, protein–protein interactions, and cellular localization. It can lead to conformational changes in proteins that either activate or inhibit their enzymatic and binding activities, impacting a broad array of cellular processes.^[^ ^79^ ^]^

## 4 PTMs of RNA Modifying Proteins

PTMs and RNA modifying proteins play pivotal roles in cellular biology, each fundamentally influencing gene expression and cellular function. The PTMs of RNA modifying proteins are especially significant as they fine‐tune the activities of these proteins, directly impacting the processing and function of RNA. These modifications not only modulate RNA metabolism in response to cellular cues and stress but also contribute to the dynamic regulation of gene expression essential for development and disease responses. As such, understanding the PTMs of RNA modifying proteins opens a window to deciphering complex regulatory networks that underpin cellular homeostasis and pathological transformations.

### 4.1 Methylation of RNA‐Modifying Proteins in Regulating Cellular Processes and Disease Progression

**Figure 5:**
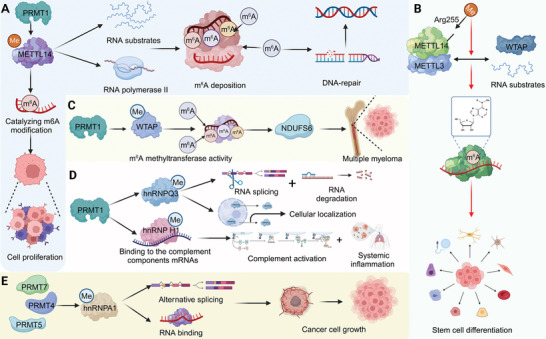
Roles of methylation on RNA‐modifying proteins in regulating cellular processes and disease progression. A) PRMT1 interacts with the METTL3‐METTL14 complex to catalyze m^6^A modification on RNA substrates. This modification is facilitated by RNA polymerase II and involves the deposition of m^6^A marks on RNA, which plays a crucial role in DNA repair. The m^6^A modification is linked to cell proliferation, highlighting its significance in cancer development. B) The METTL3‐METTL14 complex, along with WTAP, methylates RNA substrates. The Arg255 residue is crucial for the catalytic activity of this complex. m^6^A methylation influences stem cell differentiation and various cellular functions by modulating RNA stability and translation. C) PRMT1 enhances m^6^A methyltransferase activity through its interaction with WTAP, affecting *NDUFS6* mRNA. This modification plays a role in multiple myeloma, illustrating the connection between m^6^A methylation and cancer. D) PRMT1‐mediated methylation of hnRNP proteins, such as hnRNPQ3 and hnRNPH1, influences RNA splicing and degradation. This process affects cellular localization, complement activation, and systemic inflammation, demonstrating the broad impact of RNA methylation on cellular and immune functions. E) PRMT4 and PRMT5 modulate the methylation of hnRNPA1, affecting alternative splicing and RNA binding. This regulation is crucial for cancer cell growth, emphasizing the role of methylation on RNA‐modifying proteins in controlling oncogenic processes.

Methylation of RNA modifying proteins is pivotal in maintaining normal physiological functions and genetic integrity. This PTM, primarily mediated by PRMTs, is essential for modulating the activity, localization, and interaction of key RNA modifying proteins. Aberrant methylation can disrupt these critical functions, leading to a cascade of cellular dysfunctions and contributing to the pathogenesis of various diseases, including cancer (**Figure** **5**). For instance, methylation of METTL14 at arginine 255 by PRMT1 enhances the METTL3/METTL14 complex's interaction with WTAP and its RNA substrates, boosting global mRNA m^6^A levels. This arginine methylation is crucial for mouse embryonic stem cell differentiation, particularly into endoderm lineages.^[^ ^80^ ^]^ Similarly, PRMT1‐mediated arginine methylation of METTL14 enhances its interaction with RNA substrates and RNA polymerase II, boosting its RNA methylation activity and influencing m^6^A deposition in mammalian cells. This modification is critical for stem cell maintenance and DNA repair.^[^ ^81^ ^]^ In the context of cancer, PRMT1 silencing inhibits multiple myeloma cell proliferation and tumorigenesis by reducing oxidative phosphorylation via NDUFS6 downregulation. PRMT1‐mediated methylation of WTAP modulates its m^6^A methyltransferase activity, influencing NDUFS6 expression. Combining PRMT1 inhibitors with bortezomib shows synergistic effects against multiple myeloma progression.^[^ ^50^ ^]^ Furthermore, PRMT1 catalyzes critical arginine methylation at the C‐terminus of METTL14, enhancing its ability to bind RNA and catalyze m^6^A modification of mRNA. This PTM not only boosts METTL14's enzymatic function but also promotes cell proliferation, implicating a novel regulatory mechanism of m^6^A methylation in tumorigenesis.^[^ ^82^ ^]^

In addition to methylation of RNA writer protein, PRMTs extend their enzymatic activity to RNA reader proteins. For instance, PRMT1 interacts with and methylates hnRNPQ3, influencing RNA metabolism steps such as splicing and degradation. Methylation targets hnRNPQ3's C‐terminal RGG box, crucial for its nuclear localization. Inhibiting methylation shifts hnRNPQ3 from the nucleus to the cytoplasm, underscoring the functional significance of its methylation in cellular localization.^[^ ^83^ ^]^ In addition, PRMT1 regulates liver biology and protects against alcohol‐induced liver injury by methylating hnRNP H1. This methylation reduces hnRNP H1's binding to mRNAs of key complement components, suppressing their expression. In PRMT1 knockout mice, reduced methylation leads to increased complement activation and systemic inflammation in multiple organs.^[^ ^84^ ^]^ Furthermore, PRMT7, along with PRMT4 and PRMT5, methylates hnRNPA1, influencing its RNA binding and alternative splicing functions. This modification is closely linked with spliceosome and RNA‐related pathways, and is prevalent in cancer cells where it correlates with abnormal splicing and enhanced cell growth.^[^ ^85^ ^]^

### 4.2 Phosphorylation of RNA‐Modifying Proteins in Regulating Cellular Processes and Disease Progression

#### 4.2.1 Phosphorylation of RNA Writer Proteins

**Figure 6:**
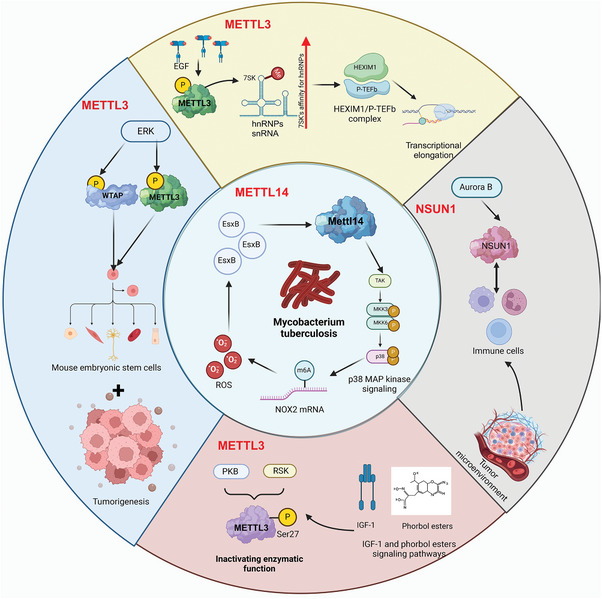
Phosphorylation of METTL3, METTL14, and NSUN1 in regulating cellular processes and disease development. METTL3, in complex with WTAP and phosphorylated by ERK signaling, is crucial in mouse embryonic stem cell differentiation and tumorigenesis. In addition, METTL3 interacts with hnRNPs and snRNA, influencing transcriptional elongation through the HEXIM1/P‐TEFb complex. Phosphorylation of METTL3 by EGF increases its affinity for hnRNPA, promoting transcriptional regulation. Moreover, METTL3 phosphorylation at Ser27 by PKB and RSK inactivates its enzymatic function, linking it to IGF‐1 and phorbol ester signaling pathways, which impact various cellular functions. METTL14 regulates the expression of *NOX2* mRNA via m^6^A modification, affecting ROS production and p38‐MAPK signaling, which is crucial for the immune defense against *Mycobacterium tuberculosis*. The bacterium secreted EsxB disrupts this methylation by inhibiting METTL14 phosphorylation. NSUN1, phosphorylates by Aurora B, plays a significant role in immune cell function and the tumor microenvironment.

Phosphorylation of RNA writer proteins is a pivotal mechanism that modulates their enzymatic activities, influencing crucial biological processes such as RNA methylation and miRNA maturation. This PTM can alter protein function, localization, and interactions within the cell, thereby directly affecting the epigenetic landscape and gene regulation (**Figure** **6**). For instance, phosphorylation of METTL3, triggered by epidermal growth factor signaling, plays a critical role in enhancing its enzymatic activity, which is crucial for the methylation of small nuclear RNA 7SK. This PTM significantly boosts 7SK's affinity for hnRNPs, thereby facilitating the release of the HEXIM1/P‐TEFb complex. This release is essential for promoting transcriptional elongation. This pathway exemplifies a pivotal role for METTL3 phosphorylation in mediating the connection between extracellular signals and transcriptional dynamics.^[^ ^86^ ^]^ In addition, ATM‐mediated phosphorylation of METTL3 at S43 activates its localization to DNA damage sites, where it facilitates methylation of DNA damage‐associated RNAs. This methylation recruits YTHDC1, enhancing the formation of DNA‐RNA hybrids necessary for RAD51 and BRCA1 to execute homologous recombination repair.^[^ ^87^ ^]^ Moreover, ERK phosphorylates METTL3 and WTAP at specific serine residues, enhancing the stability of the m^6^A methyltransferase complex via deubiquitination by USP5. This phosphorylation not only maintains the pluripotency of mouse embryonic stem cells by preventing decay of m^6^A‐marked transcripts but also contributes to tumorigenesis in ERK‐activated human cancer cells.^[^ ^88^ ^]^ Interestingly, sevoflurane anesthesia in elderly mice leads to a reduction in METTL3 phosphorylation via MAPK/ERK pathway suppression, affecting its RNA‐binding efficiency. This alteration in PTM results in widespread changes in m^6^A methylation in the hippocampus, notably disrupting methylation in the 5′UTRs of key genes. These modifications are implicated in the pathogenesis of postoperative cognitive dysfunction, underscoring the critical role of METTL3 phosphorylation in cognitive integrity post‐surgery.^[^ ^89^ ^]^ Coilin, a Cajal body marker, is essential for miRNA biogenesis by modulating METTL3 phosphorylation. Suppression of coilin reduces m^6^A methylation on miRNAs, lowers METTL3 levels, and disrupts its phosphorylation, thereby impairing its complex formation with METTL14 and WTAP. This underscores the vital role of coilin in the phosphorylation‐dependent regulation of METTL3 during miRNA maturation.^[^ ^90^ ^]^ Furthermore, a newly identified subset of C5aR1‐positive neutrophils in breast cancer promotes tumor glycolysis and progression by enhancing ENO1 expression. These neutrophils upregulate ERK1/2 signaling, leading to phosphorylation and stabilization of WTAP, which increases m^6^A methylation of *ENO1* mRNA.^[^ ^91^ ^]^ In NASH progression, hepatic WTAP plays a crucial role in regulating ectopic lipid accumulation and inflammation. CDK9‐mediated phosphorylation of WTAP drives its translocation from the nucleus to the cytosol, a pivotal modification that significantly influences WTAP's regulatory functions in NASH.^[^ ^92^ ^]^ In addition, IFN‐γ activates ERK signaling, leading to WTAP phosphorylation, which enhances m^6^A modification of immunosuppressive molecules like IDO1, PD‐L1, ICAM1, and VCAM1 in mesenchymal stem cells (MSCs). This PTM of WTAP boosts MSC‐mediated immunosuppression and improves therapeutic outcomes in colitis and arthritis models.^[^ ^93^ ^]^

Emerging evidence indicates that the phosphorylation of METTL proteins plays a pivotal role in infectious disease processes. For example, METTL14 mediated m^6^A methylation is crucial for the immune defense against *Mycobacterium tuberculosis*. The bacterium secretes EsxB, which disrupts this methylation by inhibiting METTL14 phosphorylation via interference with p38 MAP kinase signaling. This inhibition suppresses the m^6^A modification of NOX2 mRNA, reducing ROS production and enhancing bacterial survival. Targeting this pathway may offer a strategy for developing immunomodulators against tuberculosis.^[^ ^94^ ^]^ Similarly, TBK1 phosphorylates METTL3 at serine 67, activating its role in antiviral defense by enhancing mRNA stability and protein translation. This PTM facilitates increased translation within the antiviral response, stabilizing *IRF3* mRNA and bolstering type I interferon production.^[^ ^95^ ^]^ Interestingly, alphaherpesvirus kinases, particularly through the viral US3 protein, phosphorylate components of the m^6^A methyltransferase complex. This phosphorylation inhibits the complex's activity, drastically reducing m^6^A levels in mRNA from virus‐infected cells. Although not crucial for viral replication in culture, this strategy suggests an evolutionary adaptation to minimize methylation of viral transcripts, highlighting a novel regulatory mechanism of mRNA modification by phosphorylation.^[^ ^96^ ^]^

The METTL1‐WDR4 complex, critical for tRNA stability and cancer development, relies on WDR4 to scaffold METTL1 and tRNA interaction. Phosphorylation at S27 in METTL1's N‐terminal region, previously considered disordered, inhibits its methyltransferase activity by disrupting the catalytic center. This finding underscores the significance of phosphorylation in regulating METTL1‐WDR4 activity.^[^ ^97^ ^]^ In addition, METTL1 is phosphorylated at Ser27 by PKB and RSK, a process regulated by signaling pathways activated by IGF‐1 and phorbol esters. This phosphorylation inactivates METTL1's enzymatic function, as evidenced by the inability of phosphorylation‐mimicking mutants (S27D, S27E) to complement growth in trm8‐deficient yeast, unlike the non‐phosphorylatable S27A mutant.^[^ ^98^ ^]^

Phosphorylation of the NSUN family of RNA methyltransferases is a crucial regulatory mechanism that links cellular signaling to RNA metabolism, influencing disease progression and therapeutic responses. This modification acts as a fundamental mediator, converting external and internal signals into functional changes in RNA dynamics, affecting critical processes such as cancer progression and vascular health. For instance, NSUN1 phosphorylation not only influences its function but also its interaction with immune cells, underscoring its role in immune response within the tumor microenvironment.^[^ ^99^ ^]^ Notably, Aurora B‐mediated phosphorylation of NSUN2 suppresses its methyltransferase activity, which is essential for regulating ICAM‐1 mRNA methylation and promoting leukocyte adhesion in vascular inflammation. However, inflammatory stimuli such as TNF‐α and homocysteine counteract this inhibition by repressing Aurora B's phosphorylation of NSUN2, thereby enhancing NSUN2's activity. This interaction highlights how inflammatory responses can modulate RNA methylation processes, directly impacting vascular health and disease progression.^[^ ^100^ ^]^ Similarly, phosphorylation of NSUN2 by Aurora B at Ser139 modulates its distribution and interaction with nucleolar proteins during mitosis. This modification also suppresses NSUN2's methyltransferase activity, influencing the assembly of the nucleolar RNA‐processing machinery.^[^ ^101^ ^]^ Moreover, phosphorylation of NSUN2 and METTL1, regulated by Aurora B and Akt respectively, modulates their tRNA methyltransferase activity, influencing cellular sensitivity to 5‐fluorouracil (5‐FU) in HeLa cells. Dephosphorylation of these enzymes enhances their activity, reducing 5‐FU sensitivity. This connection highlights targeting phosphorylation of NSUN2 and METTL1 as a novel approach to potentiate chemotherapeutic efficacy.^[^ ^102^ ^]^ Furthermore, CDK13‐mediated phosphorylation at Ser327 activates NSUN5, leading to the m^5^C methylation of *ACC1* mRNA, which increases its stability and nuclear export via ALYREF. This mechanism boosts ACC1 expression, fostering aberrant lipid metabolism and cancer progression (**Table** **1**).^[^ ^103^ ^]^

**Table 1:** Regulatory mechanisms of phosphorylation modifications in RNA modifying proteins.

Protein	Type	Site	Kinases	Effect	RNA substrates	Interactor	Location	Mechanism	Biological implications/Disease	Ref.
METTL3	Writer	∖	EGF	Activation	snRNA 7SK	∖	Nucleus	Boosting 7SK's affinity for hnRNPs	Promoting transcriptional elongation	[86]
METTL3	Writer	S43	ATM	Activation	DNA damage‐associated RNAs	YTHDC1	Nucleus	Recruiting YTHDC1 for protection	Homologous recombination repair	[87]
METTL3 WTAP	Writer	Serines	ERK	Activation	Pluripotent factor transcripts	USP5	Nucleus	Promoting m^6^A‐labeled transcripts decay	Maintaining mESC pluripotency	[88]
METTL3	Writer	∖	MAPK/ERK	Activation	BDNF, SOX2, SYN1	∖	∖	Perturbation in m^6^A methylation	Postoperative cognitive dysfunction	[89]
WTAP	Writer	S341	ERK1/2	Activation	ENO1	∖	∖	Enhancing protein stability	Breast cancer	[91]
WTAP	Writer	∖	CDK9	Activation	IGFBP1, CD36, CCL2	HDAC1	Cytosol	Translocation from nucleus to cytosol	Influencing NASH progression	[92]
METTL14	Writer	T72	EsxB	Inhibition	Nox2	IGF2BP1	∖	Disrupting m^6^A methylation	Enhancing *Mycobacterium tuberculosis* survival	[94]
METTL3	Writer	S67	TBK1	Activation	IRF3	∖	∖	Enhancing mRNA stability and protein translation	Activating antiviral defense	[95]
METTL1	Writer	S27	∖	Activation	tRNAs	WDR4	∖	Disrupting catalytic center	Regulating METTL1/WDR4 activity	[97]
NSUN2	Writer	∖	AURKB	Activation	ICAM‐1	TNF‐α, HCY	Nucleus	Suppressing methyltransferase	Vascular inflammation and allograft arteriosclerosis	[100]
NSUN2	Writer	S139	AURKB	Activation	Nuclear RNA	NPM1	Nucleus	Suppressing methyltransferase	Nucleolar RNA‐processing machinery assembly	[101]
NSUN5	Writer	S327	CDK13	Activation	*ACC1* mRNA	ALYREF	Nucleus	Enhancing m^5^C methylation	Cancer progression	[103]
ALKBH5	Eraser	∖	SRC	Activation	Nuclear mRNA	YTHDF2	Nucleus	Suppressing m^6^A modification	Protecting against ferroptosis	[104]
ALKBH5	Eraser	∖	LATS2	Activation	*LATS2* mRNA	∖	∖	Enhancing stability and nuclear retention	Promoting glioblastoma	[105]
FTO	Eraser	∖	GSK‐3	Activation	mRNA	∖		Modulating m^6^A methylation	Regulating stem cell pluripotency	[106]
FTO KLF5	Eraser	∖	GSK3‐β	Activation	*MYC* mRNA	∖	∖	Promoting ubiquitination	Increasing cardiomyocyte apoptosis	[107]
FTO	Eraser	∖	PKC‐β	Activation	∖	∖	∖	Enhancing FTO stability	Influencing metabolic diseases	[108]
FTO	Eraser	S256	∖	Activation	∖	∖	∖	Reducing demethylase activity	Regulation of FTO function	[109]
IGF2BP1	Reader	S181	mTORC2	Activation	*MYC* mRNA	∖	Cytosol	Enhancing *MYC* translation	Decreasing cell apoptosis	[110]
hnRNP A0	Reader	∖	∖	Activation	mRNA	∖	∖	Promoting mitosis	Influencing colorectal cancer	[111]
hnRNP L	Reader	∖	Akt	Activation	HPV16 mRNA	U2AF65 SAM68	∖	Diminishing HPV16 mRNA binding	Influencing viral gene expression	[112]

#### 4.2.2 Phosphorylation of RNA Demethylase Enzymes

Phosphorylation of RNA demethylase enzymes, such as ALKBH5 and FTO, represents a critical regulatory mechanism that integrates signaling pathways with epigenetic controls to influence cellular physiology and disease progression. These modifications alter the stability, localization, and activity of the demethylases, thereby modulating the methylation status of RNA and affecting gene expression profiles crucial for various biological outcomes. For instance, EGFR signaling suppresses m^6^A modification in glioblastoma by phosphorylating ALKBH5 via Src kinase, which prevents ALKBH5's CRM1‐mediated nuclear export, enhancing m^6^A demethylation in the nucleus. This action helps protect against ferroptosis by regulating m^6^A levels on *GCLM* mRNA via YTHDF2, establishing an EGFR‐ALKBH5‐GCLM axis. Targeting this pathway enhances the efficacy of EGFR and GCLM inhibitors, suggesting a new therapeutic strategy against aggressive cancers.^[^ ^104^ ^]^ Similarly, LATS2 phosphorylates ALKBH5, enhancing its stability and nuclear retention, which in turn demethylates m^6^A from *LATS2* mRNA, stabilizing the transcript. This feedback loop promotes glioblastoma progression, indicating a non‐canonical Hippo pathway role in RNA processing.^[^ ^105^ ^]^

GSK‐3 regulates stem cell pluripotency by modulating the m^6^A methylation pathway through phosphorylation of FTO. Impaired GSK‐3 activity leads to reduced FTO polyubiquitination and increased FTO stability, resulting in decreased m^6^A levels on specific mRNAs. This novel mechanism underscores the intersection of GSK‐3 signaling and mRNA methylation in controlling stem cell pluripotency.^[^ ^106^ ^]^ Similarly, in myocardial ischemia/reperfusion injury models, GSK3β enhances phosphorylation of FTO and KLF5, promoting their ubiquitination and subsequent degradation. This degradation process results in reduced Myc expression, increasing cardiomyocyte apoptosis and oxidative stress.^[^ ^107^ ^]^ In addition, PKCβ enhances FTO stability by inhibiting its ubiquitin‐proteasome degradation and phosphorylating it on threonine. This regulation of FTO by PKCβ facilitates adipogenesis and may influence obesity and metabolic disease progression. PKCβ’s inhibition also suppresses differentiation in FTO‐overexpressing cells, underscoring a novel pathway that links these proteins in the regulation of adiposity and metabolic health.^[^ ^108^ ^]^ Interestingly, phosphorylation at serine 256 reduces the demethylase activity of FTO, a modification observed only in baculovirus‐expressed samples, not in those expressed in *E. coli*. The presence of calcium slightly stabilizes FTO, albeit at the cost of reduced catalytic efficiency. Both expression systems confirm FTO's ability to form homodimers, with structural analysis supported by SAXS data, underscoring the complex regulation of FTO function by PTMs and cofactors.^[^ ^109^ ^]^

#### 4.2.3 Phosphorylation of RNA Reader Proteins

Phosphorylation of RNA reader proteins serves as a dynamic regulatory mechanism that intricately integrates cellular signaling to RNA metabolism, influencing key physiological processes and disease outcomes. For instance, phosphorylation of IGF2BP1 plays a pivotal role in regulating its function and the stability of *c‐MYC* mRNA. mTORC2‐mediated Ser181 phosphorylation of IGF2BP1 enhances *c‐MYC* translation, while inhibition of this phosphorylation destabilizes *c‐MYC* mRNA, increasing apoptosis. In addition, preventing Tyr396 phosphorylation by Src kinase leads to the sequestration of transcripts by IGF2BP1, reducing their translation. Combining mTORC2 and Src inhibitors synergistically suppresses tumor growth, indicating a potential targeted therapy for malignancies overexpressing IGF2BP1.^[^ ^110^ ^]^ In addition, phosphorylation of hnRNP A0 significantly influences its role in promoting mitosis via the RAB3GAP1‐ZWINT1 cascade in colorectal cancer cells. Differential interaction of phosphorylated hnRNP A0 with mRNAs in cancer versus non‐tumorous cells underscores its potential as a target for tumor treatment, highlighting the critical impact of phosphorylation on its tumor‐specific functions.^[^ ^111^ ^]^ Moreover, Akt kinase inhibition disrupts hnRNP L phosphorylation, diminishing its binding to HPV16 mRNAs, and subsequently enhances HPV16 late gene expression through altered RNA processing. This regulation involves shifts in RNA binding protein interactions, including increased U2AF65 and Sam68 association with HPV16 mRNAs. The manipulation of hnRNP L binding sites further activates HPV16 late gene expression, underscoring hnRNP L phosphorylation as critical for the temporal control of viral gene expression.^[^ ^112^ ^]^

### 4.3 Acetylation of RNA‐Modifying Proteins in Regulating Cellular Processes and Disease Progression

**Figure 7:**
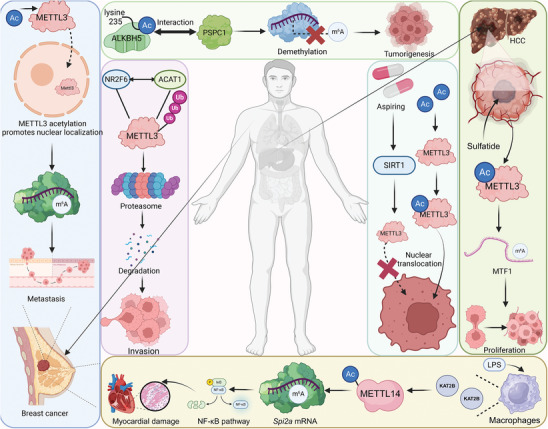
Mechanisms and impacts of RNA‐modifying protein acetylation in cancer and cellular processes. Acetylation of METTL3 promotes its nuclear localization, which enhances m^6^A modification on RNA and contributes to metastasis in breast cancer. This modification facilitates cancer cell invasion and progression. In addition, lysine 235 acetylation of ALKBH5 disrupts its interaction with PSPC1, preventing m^6^A demethylation and leading to tumorigenesis. Notably, NR2F6 and ACAT1 mediate acetylation‐related stabilization of METTL3 regulates cellular invasion and metastasis. SIRT1 deacetylates METTL3, modulating its nuclear translocation and activity. Aspirin promotes this deacetylation, affecting cancer cell proliferation and tumor progression. Sulfatide‐induced acetylation of METTL3 enhances m^6^A modification of *MTF1*, promoting cancer cell proliferation. LPS boosts KAT2B in macrophages, acetylating METTL14 at K398. This ups Spi2a m6A methylation, curbing NF‐κB, cytokines, and heart damage.

Acetylation of RNA‐modifying enzymes significantly impacts cellular physiology by altering their stability and function. This modification affects RNA writers, erasers, and readers, influencing RNA metabolism and gene expression. Understanding these dynamics is crucial for identifying potential therapeutic targets in diseases linked to RNA modification dysregulation (**Figure** **7**). For instance, NR2F6 activates transcription of ACAT1, which interacts with and stabilizes METTL3 by preventing its ubiquitin‐proteasome‐mediated degradation. This acetylation‐related stabilization of METTL3 by ACAT1 suppresses TNBC cell migration and invasion, highlighting how the NR2F6/ACAT1/METTL3 axis plays a crucial role in reducing tumor aggressiveness.^[^ ^113^ ^]^ In addition, acetylation controls METTL3 localization and function, with nuclear METTL3 promoting breast cancer metastasis through an IL‐6‐dependent mechanism that enhances m^6^A methylation. Conversely, SIRT1 inhibition, augmented by aspirin, blocks METTL3's nuclear translocation, reducing metastasis by curtailing m^6^A activity. An acetylation‐mimetic METTL3 variant disrupts this process, leading to diminished metastatic capacity, suggesting that METTL3's acetylation state is pivotal in modulating its oncogenic potential in breast cancer. Moreover, sulfatide exposure in HCC cells leads to METTL3 acetylation, reducing its interaction with *MTF1* mRNA and the METTL14‐WTAP complex. This PTM diminishes m^6^A methylation of *MTF1* mRNA, thereby increasing its stability and expression. Elevated MTF1 levels enhance HCC cell proliferation and correlate with poor prognosis. This highlights acetylation's role in modulating METTL3 activity and its impact on tumor progression through *MTF1* mRNA stability.^[^ ^114^, ^115^ ^]^ Notably, LPS increases KAT2B in macrophages, leading to acetylation and stabilization of METTL14 at K398. This acetylation enhances METTL14's m^6^A methylation of Spi2a, inhibiting the NF‐κB pathway and reducing cytokine release and myocardial damage. This highlights the critical role of METTL14 acetylation in regulating macrophage activation and sepsis progression.^[^ ^115^ ^]^

Acetylation not only affects RNA writer proteins but can also extend to RNA eraser and reader proteins, impacting their functions in RNA metabolism. For instance, acetylation at lysine 235 enhances ALKBH5's m^6^A demethylation activity, a modification mediated by lysine acetyltransferase 8 and reversed by histone deacetylase 7. This PTM boosts ALKBH5's interaction with PSPC1, increasing its affinity for m^6^A‐modified mRNA, thereby promoting m^6^A removal. Elevated in cancers, K235 acetylation of ALKBH5 contributes to tumorigenesis, revealing a targetable mechanism in cancer therapy.^[^ ^116^ ^]^ In addition, SIRT1‐mediated deacetylation of IGF2BP2 regulates its interaction with the *ATP6V1A* mRNA, influencing RNA stability and cellular lysosomal function. In breast cancer cells, low SIRT1 levels lead to IGF2BP2 acetylation, enhancing transcript degradation via XRN2 recruitment, thereby impairing v‐ATPase complex expression and lysosomal activity. This dysfunction increases exosome secretion, promoting tumor survival and invasiveness. This mechanism underscores the critical impact of IGF2BP2's acetylation state on its functional role in cancer pathophysiology.^[^ ^117^ ^]^

### 4.4 SUMOylation of RNA‐Modifying Proteins in Regulating Cellular Processes and Disease Progression

SUMOylation critically regulates RNA modifying proteins, impacting their stability, activity, and interactions. This modification alters mRNA methylation dynamics, affecting gene expression and cellular responses to stress. By fine‐tuning the function of key enzymes like METTL3 and ALKBH5, SUMOylation plays a pivotal role in both normal cellular functions and disease progression, highlighting its potential as a therapeutic target.

**Figure 8:**
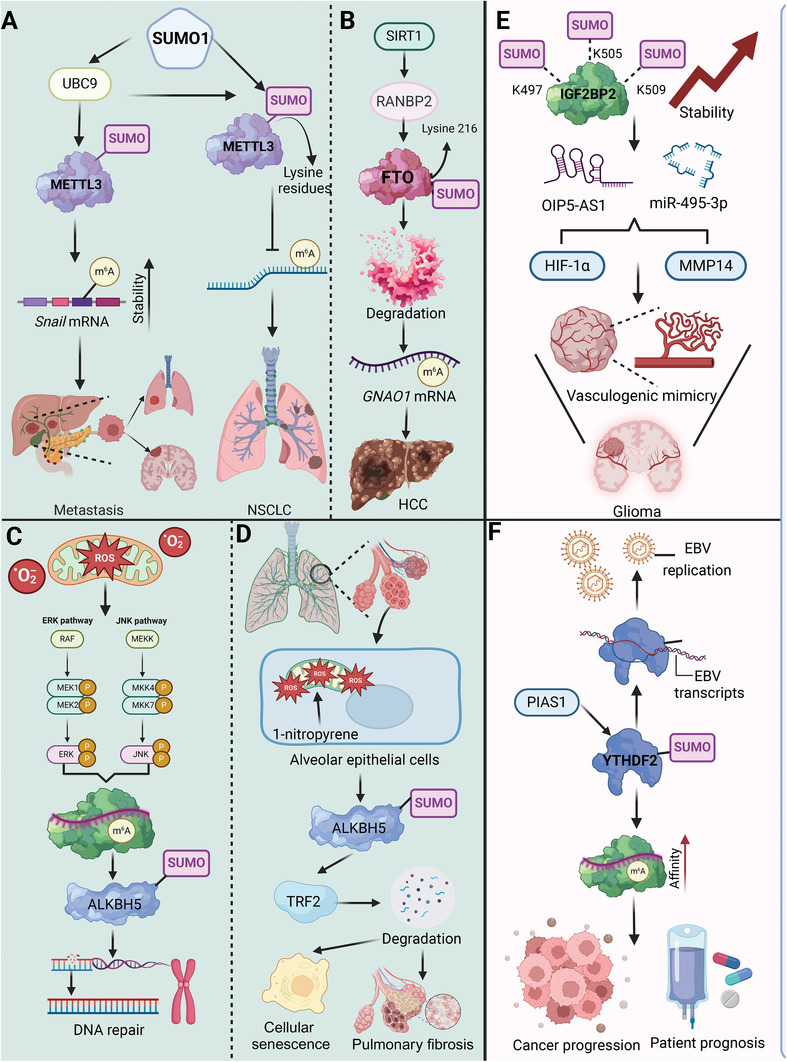
The effects of RNA‐modifying protein SUMOylation in regulating cellular processes and disease progression. A) The SUMOylation of METTL3 by UBC9 enhances its stability and activity. SUMOylated METTL3 increases the stability of *Snail* mRNA through m^6^A modification, promoting metastasis in various cancers, including NSCLC. B) RANBP2‐mediated SUMOylation of FTO at lysine 216, regulated by SIRT1, leads to its degradation. This affects the m^6^A demethylation of *GNAO1* mRNA, contributing to HCC progression. C) ALKBH5 SUMOylation, influenced by ROS and the ERK/JNK pathways, plays a crucial role in DNA repair mechanisms. This modification affects ALKBH5's stability and function, essential for maintaining genomic integrity. D) 1‐Nitropyrene induces pulmonary fibrosis by promoting ROS generation in alveolar epithelial cells, leading to SUMOylation of ALKBH5 and subsequent TRF2 degradation, driving cellular senescence and fibrosis. E) SUMOylation stabilizes IGF2BP2 by modifying lysine residues K497, K505, and K509. This enhances its interaction with lncRNAs (OIP5‐AS1 and miR‐495‐3p), promoting vasculogenic mimicry in glioma. F) PIAS1‐mediated SUMOylation of YTHDF2 increases its affinity for m^6^A‐modified EBV transcripts, facilitating EBV replication and contributing to cancer progression. This modification is associated with poor patient prognosis.

SUMOylation of METTL3 plays a crucial role in regulating cellular m^6^A dynamics and the resulting biological outcomes. The enhancement or reduction of its methylation activity is closely correlated with specific SUMOylation sites (**Figure** **8**). For instance, SUMOylation of METTL3, mediated by SUMO1 and enhanced by UBC9 upregulation upon mitogen stimulation, plays a critical role in HCC progression. This PTM enhances METTL3's ability to regulate *Snail* mRNA stability through m^6^A methylation, linking METTL3 SUMOylation to increased metastatic potential in liver cancer.^[^ ^118^ ^]^ Similarly, METTL3 is upregulated and modulates circ_0000677 via m^6^A modification, enhancing circ_0000677′s influence on cell proliferation and drug resistance through ABCC1. METTL3 itself is regulated by SUMO1‐mediated SUMOylation. A mutation in METTL3 (K459) attenuates CRC progression by disrupting the circ_0000677/ABCC1 axis. On the contrary, METTL3 undergoes SUMOylation at specific lysine residues, which, contrary to affecting its stability or localization, selectively represses its methylation activity. This modification decreases m^6^A levels in mRNAs, impacting gene expression and tumorigenic potential in NSCLC cells.^[^ ^119^ ^]^ Notably, SARS‐CoV‐2 infection enhances METTL3 expression and alters its cellular distribution, interacting with the viral RdRp. This interaction appears to regulate METTL3's SUMOylation and ubiquitination, impacting its role in viral RNA m^6^A modification and influencing SARS‐CoV‐2 replication. This highlights a dynamic interplay between host PTM machinery and viral proteins, crucial for viral life cycle regulation.^[^ ^120^ ^]^ Moreover, the stability and nuclear localization of NSUN2 are elevated by SUMO‐2/3‐mediated post‐translational modifications, enhancing its carcinogenic activities. These insights suggest that modulating NSUN2's SUMOylation could offer new therapeutic avenues for gastric cancer.^[^ ^121^ ^]^

Interestingly, ROS seem to play a pivotal role in modulating cellular functions through the SUMOylation of ALKBH5. For instance, ROS‐induced stress enhances mRNA m^6^A levels via ERK/JNK signaling that promotes SUMOylation of the ALKBH5, inhibiting its activity and enhancing gene expression for DNA repair. This regulatory mechanism protects genomic integrity, particularly in hematopoietic stem/progenitor cells, revealing a critical link between ALKBH5 SUMOylation and maintenance of genome stability under oxidative stress.^[^ ^122^ ^]^ In addition, exposure to 1‐nitropyrene in alveolar epithelial cells triggers mitochondrial ROS overproduction, leading to ALKBH5 SUMOylation and its subsequent proteasomal degradation. This cascade enhances FBXW7's m^6^A modification, increasing its ubiquitin ligase activity toward TRF2, promoting TRF2 degradation, cellular senescence, and pulmonary fibrosis. Mitigation of these effects by Mito‐TEMPO underscores the potential for targeted interventions against 1‐nitropyrene induced pulmonary fibrosis through modulation of ALKBH5 SUMOylation.^[^ ^123^ ^]^ In addition to impacting ALKBH5, SUMOylation also significantly influences the stability of FTO. SIRT1 promotes HCC progression by activating RANBP2, which induces SUMOylation and subsequent degradation of FTO at lysine 216. This modification reduces FTO levels, leading to increased m^6^A modification of the tumor suppressor GNAO1, thereby decreasing its mRNA expression.^[^ ^124^ ^]^ Interestingly, arsenic exposure increases FTO SUMOylation at lysine‐216, decreasing FTO levels in lung tissue and enhancing oxidative damage via an m6A‐dependent pathway mediated by IGF2BP3. This PTM of FTO underscores a novel mechanism linking m^6^A dynamics to oxidative stress, offering potential therapeutic targets for oxidative damage.^[^ ^125^ ^]^

The SUMOylation of RNA reader proteins is not only essential for ensuring their stability, but also increasing their ability to specifically recognize and bind to m^6^A sites, facilitating efficient RNA processing and regulation. For example, SUMOylation at lysine residues K497, K505, and K509 enhances IGF2BP2 stability by inhibiting its degradation via the ubiquitin‐proteasome pathway. This modification upregulates IGF2BP2 in glioma, fostering VM (vasculogenic mimicry) formation by stabilizing OIP5‐AS1 and disrupting miR‐495‐3p's inhibition of HIF‐1α and MMP14, critical VM components. This mechanism suggests targeting IGF2BP2 SUMOylation could offer new therapeutic approaches for glioma.^[^ ^126^ ^]^ Similarly, YTHDF2 is SUMOylated at lysine 571, a modification that enhances its affinity for m^6^A‐modified mRNAs without affecting its ubiquitination or localization. This enhanced binding leads to altered gene expressions that contribute to cancer progression. In lung adenocarcinoma, high levels of YTHDF2 and SUMO1 correlate with poor patient prognosis, underscoring the significance of YTHDF2 SUMOylation in cancer regulation and potential therapeutic targeting.^[^ ^127^ ^]^ Interestingly, PIAS1 enhances the antiviral function of YTHDF2 by promoting its SUMOylation at specific lysine residues, which is crucial for limiting Epstein‐Barr virus (EBV) replication by reducing YTHDF2's binding to EBV transcripts, thus increasing their stability. This SUMOylation also extends to YTHDF1 and YTHDF3, demonstrating a broader role for PIAS1 in regulating the antiviral activity of the YTHDF protein family against EBV through post‐translational modifications.^[^ ^132^ ^]^

### 4.5 Ubiquitination of RNA‐Modifying Enzymes in Regulating Cellular Processes and Disease Progression

#### 4.5.1 Ubiquitination of RNA Writer Proteins

**Figure 9:**
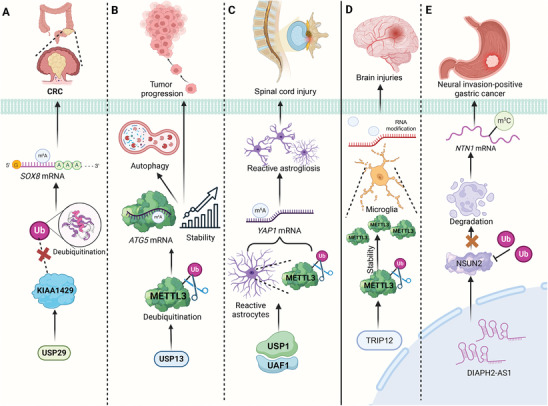
The effects of writer ubiquitination in regulating cellular processes and disease progression. A) KIAA1429‐mediated deubiquitination by USP29 stabilizes METTL3, enhancing m^6^A modification on *SOX8* mRNA. This modification promotes CRC progression by increasing the stability and translation of *SOX8* mRNA. B) USP13 deubiquitinates METTL3, stabilizing *ATG5* mRNA through m^6^A modification. This regulation enhances autophagy, contributing to tumor progression by maintaining cellular homeostasis under stress conditions. C) USP1 and UAF1 mediate METTL3 deubiquitination, which increases m^6^A modification on *YAP1* mRNA. This modification promotes reactive astrogliosis and the activation of reactive astrocytes, playing a crucial role in the response to spinal cord injury. D) TRIP12‐mediated stabilization of METTL3 in microglia, due to reduced K48‐linked polyubiquitination, amplifies neuroinflammation and contributes to brain injury. E) NSUN2 ubiquitination leads to the degradation of m^5^C‐modified *NTN1* mRNA. This modification is crucial for the stability of *NTN1* mRNA, which influences neural invasion in gastric cancer. The degradation process involves the interaction of NSUN2 with DIAPH2‐AS1, affecting the metastatic potential of cancer cells.

Members of the ubiquitin specific protease (USP) family and STUB1 are crucial for maintaining the stability of RNA writer proteins, affecting various cellular functions and disease progression (**Figure** **9**). For instance, USP29 enhances malignant cell proliferation by stabilizing KIAA1429 through deubiquitination. KIAA1429, in turn, promotes the stability of *SOX8* mRNA via m^6^A modification, forming a regulatory axis that drives CRC growth. This KIAA1429/SOX8 pathway underscores a potential therapeutic target for inhibiting CRC progression.^[^ ^129^ ^]^ Similarly, USP13 upregulates and stabilizes METTL3 by removing K48‐linked ubiquitin chains, enhancing METTL3's ability to increase m^6^A levels and stabilize *ATG5* mRNA, promoting autophagy and tumor progression. Pharmacological inhibition of USP13 induces METTL3 degradation, demonstrating therapeutic efficacy in osteosarcoma.^[^ ^130^ ^]^ Following spinal cord injury, the USP1/UAF1 complex stabilizes METTL3 in reactive astrocytes by removing K48‐linked ubiquitination, enhancing m^6^A modifications on the YAP1 transcript to promote its stability and contribute to reactive astrogliosis. This highlights METTL3's pivotal role in spinal cord injury recovery through post‐translational ubiquitination, suggesting potential therapeutic avenues.^[^ ^131^ ^]^ Crucially, Elvitegravir targets METTL3 for STUB1‐mediated proteasomal degradation, disrupting its function. This presents a potential therapeutic strategy to inhibit metastasis in ESCC by controlling the degradation of METTL3. Interestingly, METTL14 stability, crucial for m^6^A RNA modification homeostasis, is regulated by METTL3 through competitive interaction with the E3 ligase STUB1, which ubiquitinates METTL14 leading to its degradation. This interaction controls m^6^A levels and impacttumorigenesis, highlighting the therapeutic potential of modulating STUB1 to manage METTL14 levels in cancer treatments.^[^ ^132^ ^]^ Moreover, METTL3 upregulation in microglia following brain injury is driven by decreased K48‐linked polyubiquitination, orchestrated by TRIP12. This reduction in METTL3 degradation amplifies inflammatory responses and exacerbates neuronal loss. Targeting this pathway offers a therapeutic approach to mitigate neuroinflammation and promote recovery in brain injuries.^[^ ^133^ ^]^

In addition to being directly regulated by the USP family and STUB1, the ubiquitination of RNA writer proteins may also be indirectly regulated. For instance, PIN1 interacts with METTL3, enhancing its stability and preventing its degradation via ubiquitin‐dependent pathways. This stabilization increases m^6^A modifications on *TAZ* and *EGFR* mRNA, boosting their translation and influencing breast cancer cell proliferation and tumor growth.^[^ ^134^ ^]^ In addition, PLAA suppresses ovarian cancer metastasis by inhibiting TRPC3‐mediated intracellular Ca^2+^ levels. Mechanistically, PLAA reduces METTL3 expression via ubiquitin‐mediated degradation, impacting *TRPC3* mRNA stability through reduced m^6^A modification.^[^ ^135^ ^]^ Moreover, cystatin A binds METTL3, promoting its ubiquitin‐proteasome‐mediated degradation, which subsequently upregulates NKX3‐1 and LHPP, leading to suppressed AKT signaling. This pathway elucidates a mechanism by which cystatin A inhibits NPC cell motility and metastasis, suggesting the cystatin A‐METTL3 axis as a potential therapeutic target for NPC.^[^ ^136^ ^]^ Furthermore, METTL14 modulates TRIB2 expression through miR‐99a‐5p, targeting *TRIB2* mRNA for degradation in ESCC. Conversely, TRIB2 promotes ubiquitin‐mediated degradation of METTL14, enhancing radioresistance and stem‐like properties.^[^ ^137^ ^]^ Importantly, non‐coding RNAs also play a critical role in regulating the ubiquitination of RNA writer proteins. DIAPH2‐AS1, a lncRNA upregulated in neural invasion‐positive gastric cancer, enhances NSUN2 stability by shielding it from ubiquitin‐proteasomal degradation at specific lysine residues. This protection facilitates the m^5^C methylation of *NTN1* mRNA by NSUN2, promoting cancer cell migration, invasion, and neural invasion.^[^ ^138^ ^]^ Similarly, circ_0001187 moderates AML progression by sponging miR‐499a‐5p, subsequently upregulating E3 ubiquitin ligase RNF113A, which promotes K48‐linked polyubiquitination and degradation of METTL3. This pathway decreases m^6^A mRNA modification, affecting AML cell proliferation and apoptosis.^[^ ^139^ ^]^

External stimuli such as infections and stress play significant roles in affecting the ubiquitination of RNA writer proteins, thereby impacting cellular responses and regulatory mechanisms. For instance, EBV infection downregulates TLR9 by modulating m^6^A methylation, where EBV‐encoded EBNA1 promotes METTL3 degradation via the ubiquitin‐proteasome pathway, reducing *TLR9* mRNA stability. In addition, YTHDF1, acting as an m^6^A reader, enhances TLR9 translation. This mechanism contributes to immune evasion in B‐cell proliferation and has implications in lymphoma.^[^ ^140^ ^]^ In addition, the ER stress response upregulates METTL14, which selectively degrades *CHOP* mRNA via m^6^A modification, shifting the cellular outcome from apoptosis to stress adaptation. This regulation involves competition with the HRD1‐ERAD system, preventing METTL14 ubiquitination and degradation.^[^ ^141^ ^]^

#### 4.5.2 Ubiquitination of RNA Eraser Proteins

**Figure 10:**
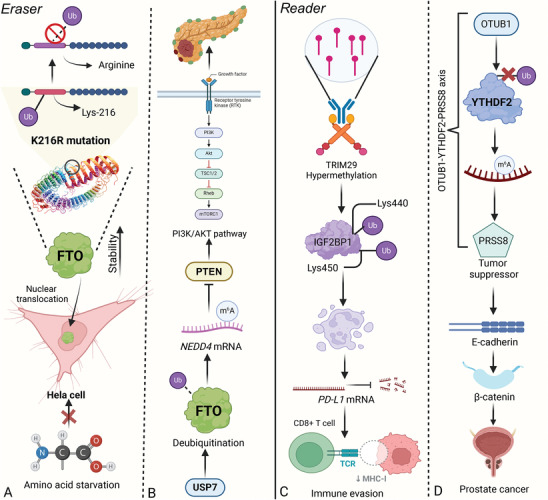
The effects of reader/eraser ubiquitination in regulating cellular processes and disease progression. A) The K216R mutation in FTO affects its ubiquitination at lysine 216, leading to increased stability and nuclear translocation under amino acid starvation conditions in HeLa cells. This modification influences FTO's ability to regulate m^6^A demethylation and impacts cellular responses to nutrient deprivation. B) USP7 deubiquitinates FTO, stabilizing it and promoting the demethylation of *NEDD4* mRNA. This regulation activates the PI3K/AKT pathway by modulating PTEN expression, influencing cancer cell growth and survival. C) Hyperubiquitination of IGF2BP1 at lysines 440 and 450, mediated by TRIM29 hypermethylation, stabilizes *PD‐L1* mRNA. This modification facilitates immune evasion by increasing PD‐L1 expression, which inhibits CD8^+^ T cell activity through MHC‐I downregulation. D) OTUB1‐mediated deubiquitination of YTHDF2 stabilizes the reader protein, enhancing its interaction with m^6^A‐modified *PRSS8* mRNA. This regulation promotes the tumor suppressor function of PRSS8, influencing E‐cadherin and β‐catenin pathways, and playing a crucial role in prostate cancer progression.

FTO ubiquitination profoundly impacts cellular functions by altering its stability and localization. Abnormal ubiquitination of FTO influences various signaling pathways crucial for metabolic regulation and disease progression (**Figure** **10**). For instance, FTO undergoes post‐translational ubiquitination at Lys‐216, crucial for its turnover and localization. In HeLa cells with a K216R mutation, ubiquitination is impaired, leading to increased FTO stability and altered phosphorylation of ribosomal S6 kinase. This mutation also restricts FTO's nuclear translocation under amino acid starvation, highlighting the role of ubiquitination in regulating FTO's cellular functions and its implications for body mass regulation.^[^ ^142^ ^]^ In addition, USP18 enhances FTO stability through de‐ubiquitination, reducing m^6^A modification of *SIRT6* mRNA, thus facilitating its expression and activating the AMPK/PGC‐1α/AKT signaling pathway. This sequence triggers mitophagy in neurocytes, offering neuroprotection in ischemic stroke models.^[^ ^143^ ^]^

In the context of cancer, FTO is significantly upregulated in gemcitabine‐resistant pancreatic cancer, with its stability enhanced by USP7‐mediated deubiquitination. FTO facilitates chemoresistance through m^6^A‐dependent demethylation of *NEDD4* mRNA, enhancing NEDD4 expression, which suppresses PTEN and activates the PI3K/AKT pathway.^[^ ^144^ ^]^ Similarly, FTO protein levels are suppressed under hypoxia via ubiquitin‐mediated degradation, facilitated by the E3 ligase STRAP at ubiquitination site K216. This reduction in FTO enhances metastasis by allowing increased stability of m^6^A‐modified *MTA1* mRNA via IGF2BP2.^[^ ^145^ ^]^ In addition, the E6E7 oncogene upregulates HK2 and increases METTL3 and WTAP expression while decreasing FTO and ALKBH5. Crucially, E6E7 also activates GSK3β, which enhances FTO's ubiquitination and proteasomal degradation. This degradation is key in modulating *HK2* mRNA processing and translation, highlighting its importance in cervical cancer pathology.^[^ ^146^ ^]^ Interestingly, IGF2BP3 upregulation promotes MIB1 expression in glioma, enhancing the ubiquitin‐proteasome‐mediated degradation of FTO. This reduction in FTO stabilizes m^6^A modifications on *CSF3* mRNA, facilitating NET (neutrophil traps) formation, which is further induced by oncolytic herpes simplex virus (HSV). BET inhibitors counteract this by blocking IGF2BP3‐induced NETosis, boosting oncolytic HSV efficacy, and suggesting a novel approach to enhance oncolytic virotherapy by targeting NETosis.^[^ ^147^ ^]^

#### 4.5.3 Ubiquitination of RNA Reader Proteins

Accumulating evidence has demonstrated that the ubiquitination of RNA reader proteins, such as IGF2BPs and YTHDF2, significantly affects cancer progression (Figure 10). For instance, in gastric cancer, TRIM29 is downregulated due to hypermethylation and enhances antitumor immunity by mediating the ubiquitination and degradation of IGF2BP1 at Lys440 and Lys450. This degradation disrupts the IGF2BP1‐mediated stabilization of *PD‐L1* mRNA, pivotal for immune evasion.^[^ ^148^ ^]^ In addition, LTN1 regulates cancer cell proliferation by ubiquitinating and destabilizing IGF2BP1, thereby inhibiting c‐Myc and IGF‐1R signaling pathways. This post‐translational modification leads to reduced IGF2BP1 levels in HCC, correlating with poor patient prognosis.^[^ ^149^ ^]^ Moreover, FBXO45 enhances liver tumorigenesis by promoting the ubiquitination and subsequent activation of IGF2BP1 at Lys190 and Lys450. This activation leads to increased PLK1 expression, driving cell proliferation and tumor growth.^[^ ^150^ ^]^ Interestingly, non‐coding RNAs appear to play a crucial role in mediating the ubiquitination of IGF2BPs. In NSCLC, circNDUFB2 enhances the ubiquitination and degradation of IGF2BPs via a complex with TRIM25, which is further potentiated by m^6^A modification of circNDUFB2. This regulatory mechanism not only suppresses IGF2BPs, a promoter of tumor progression, but also activates RIG‐I‐MAVS signaling, potentially enhancing immune response within the tumor microenvironment.^[^ ^151^ ^]^ In addition, LINRIS enhances CRC progression by stabilizing IGF2BP2 through inhibition of its ubiquitination at K139. This stabilization prevents IGF2BP2 degradation via the autophagy‐lysosome pathway, facilitating MYC‐driven glycolysis in cancer cells. Targeting the LINRIS‐IGF2BP2 axis may provide therapeutic benefits in CRC, as evidenced by reduced tumor proliferation in both orthotopic and patient‐derived xenograft models upon LINRIS inhibition.^[^ ^152^ ^]^

Importantly, YTHDF2 ubiquitination mediated by ubiquitin ligases or non‐coding RNAs is critical in regulating cancer development. For instance, YTHDF2 is upregulated in HCC and associated with poor outcomes, facilitated by decreased ubiquitination. HSP90β interaction with YTHDF2 and STUB1 in the cytoplasm inhibits STUB1‐mediated degradation of YTHDF2, enhancing YTHDF2 stability, HCC cell proliferation, and sorafenib resistance.^[^ ^153^ ^]^ In prostate cancer, OTUB1 enhances YTHDF2 stability by blocking its ubiquitination, independently of its deubiquitinase activity. YTHDF2 then decreases levels of the tumor suppressor PRSS8 by promoting its m^6^A‐dependent mRNA degradation. This OTUB1‐YTHDF2‐PRSS8 axis downregulates nuclear β‐catenin via E‐cadherin, revealing a critical pathway in prostate cancer progression and identifying potential targets for therapeutic intervention.^[^ ^154^ ^]^ Moreover, LINC00707 promotes HCC progression by enhancing cell proliferation, migration, and invasion. Mechanistically, LINC00707 interacts with YTHDF2, leading to its ubiquitination and subsequent proteasomal degradation. This interaction also impacts the cytotoxic response of NK‐92MI cells against HCC cells, suggesting that targeting LINC00707‐YTHDF2 interactions could provide therapeutic benefits in HCC.^[^ ^155^ ^]^ Similarly, increased JPX lncRNA expression exacerbates disease progression by interacting with YTHDF2 in melanoma. JPX obstructs the deubiquitination of YTHDF2 by USP10, leading to decreased YTHDF2 stability and enhanced degradation. This reduction in YTHDF2 levels allows for the stabilization of *BMP2* mRNA and subsequent activation of AKT phosphorylation, promoting melanoma cell proliferation and migration (**Table** **2**).^[^ ^156^ ^]^

**Table 2:** Ubiquitination of RNA modifying proteins and their biological implications.

Protein	Type	Modification	Site	Regulator	mRNA substrates	Enzymes	Effect of ubiquitination /deubiquitination	Mechanism	Biological implications	Refs.
KIAA1429	Writer	Deubiquitination	∖	USP29	SOX8	∖	Stabilize KIAA1429	Promoting the stability of *SOX8* mRNA through m^6^A modification	Promoting CRC malignancy	[129]
METTL3	Writer	Deubiquitination	K488	USP13	ATG5	∖	Regulate glycolytic reprogramming	Facilitating glycolysis and autophagy	Promoting tumor progression	[130]
METTL3	Writer	Deubiquitination	K48	USP1/UAF1	YAP1	∖	Inhibition of METTL3 degradation	Enhancing m^6^A modification on YAP1 transcript	Promoting motor repair	[131]
METTL14	Writer	Ubiquitination	K148 K156 K162	STUB1	EGR1	E3 ligases	Mediate METTL3 degradation	Decreasing total m^6^A levels	Affecting tumorigenesis	[132]
METTL3	Writer	Deubiquitination	∖	PIN1	TAZ/EGFR	∖	Enhance proteasomal degradation of METTL3	Increasing the m^6^A modification of *TAZ* and *EGFR* mRNA	Enhancing tumorigenesis	[134]
METTL3	Writer	Ubiquitination	∖	PLAA	TRPC3	∖	∖	Impacting *TRPC3* mRNA stability through reduced m^6^A modification	Inhibiting cancer metastasis	[135]
METTL3	Writer	Ubiquitination	∖	Cystatin A	CSTA	∖	Enhance METTL3 degradation	Upregulating NKX3‐1 and LHPP	Inhibiting NPC metastasis	[136]
METTL14	Writer	Ubiquitination	∖	TRIB2	TRIB2	COP1	Enhance proteasomal degradation of METTL14	Downregulating miR‐99a‐5p	Promoting ESCC radioresistance	[137]
NSUN2	Writer	Masking ubiquitination	K577 K579	DIAPH2‐AS1	NTN1	∖	Increase NSUN2 stability	Facilitating m^5^C methylation of *NTN1* mRNA	Enhancing tumorigenesis	[138]
METTL3	Writer	Ubiquitination	K48	circ_0001187	DOPEY2	RNF113A	Enhance METTL3 degradation	Decreasing m^6^A modification	Suppressing AML	[139]
METTL3	Writer	Ubiquitination	K48	EBNA1	TLR9	PRKN	Enhance METTL3 degradation	Downregulating *TLR9* m^6^A modification levels	Inhibiting immune escape	[140]
METTL14	Writer	Deubiquitination	∖	UPR	CHOP	HRD1	Upregulation of METTL14	Promoting *CHOP* mRNA decay through its 3′ UTR m^6^A	Increasing stress adaptation	[141]
FTO	Eraser	Ubiquitination	K216	∖	∖	∖	Mediate FTO proteasomal degradation	∖	Determining body mass and composition	^146^
FTO	Eraser	Deubiquitination	∖	USP18	SIRT6	∖	Increase FTO stability	Activating AMPK/PGC‐1α/AKT signaling and inducing mitophagy	Ameliorating nerve damage	[143]
FTO	Eraser	Ubiquitination	K216	HIF‐α	MTA1	E3 ligase	Induce FTO degradation	Increasing MTA1 expression	Promoting CRC metastasis	[145]
FTO	Eraser	Ubiquitination	∖	E6E7	HK2	∖	Decrease FTO levels	Increasing *HK2* mRNA and protein	∖	[146]
FTO	Eraser	Ubiquitination	∖	IGF2BP3	MIB1	MIB1, HERC2, HUWE1	Enhance FTO degradation	Increasing m^6^A‐mediated CSF3 release and NET formation	Enhancing oncolytic activity	[147]
IGF2BP1	Reader	Ubiquitination	K440 K450	TRIM29	TRIM29	E1, E2 and E3 ligases	Enhance IGF2BP1 degradation	Decreasing *PD‐L1* mRNA stability	Regulating antitumor T‐cell immunity in GC	[148]
IGF2BP1	Reader	Ubiquitination	∖	LTN1	mRNAs	E3 ligases	Enhance IGF2BP1 degradation	Inhibiting c‐Myc and IGF‐1R pathways	Inhibiting HCC	[149]
hnRNP A	Reader	Ubiquitination	K190 K450	FBXO45	PLK1	E3 ligases	Upregulate PLK1 expression	Activating IGF2BP1 and PLK1	Enhancing liver tumorigenesis	[150]
IGF2BP2	Reader	Deubiquitination	K139	LINRIS	MYC	∖	Block IGF2BP2 degradation	Facilitating MYC‐driven glycolysis	Enhancing CRC progression	[152]
YTHDF2	Reader	Deubiquitination	∖	HSP90β	mRNAs	STUB1	Upregulation of YTHDF2	∖	Enhancing HCC progression	[153]
YTHDF2	Reader	Inhibiting ubiquitination	∖	OTUB1	PRSS8	E2/E3 ligases	∖	Decreasing PRSS8 level	Affecting prostate cancer	[154]
YTHDF2	Reader	Ubiquitination	∖	LINC00707	∖	∖	Mediated YTHDF2 degradation	Influencing the cytotoxicity of NK‐92MI cells against HCC cells	Promoting HCC	[155]
YTHDF2	Reader	Ubiquitination	∖	lncRNA JPX	BMP2	∖	∖	Stabilizing *BMP2* mRNA and activating AKT phosphorylation	Melanoma deterioration	[156]

### 4.6 Other PTM Enzymes in Regulating Cellular Processes and Disease Progression

Recent research highlights the significant impact of PTMs such as lactylation, O‐GlcNAcylation, and S‐nitrosylation on the functionality of RNA modifying proteins. These modifications not only regulate the stability and activity of these proteins but also influence critical biological processes and disease progression through the modulation of mRNA methylation. In a model of intracerebral hemorrhage induced by hemin in PC12 cells, lactylation of METTL3 is increased, enhancing its stability and activity. This PTM upregulates m^6^A methylation of *TFRC* mRNA, promoting ferroptosis. Knockdown of METTL3 reverses these effects, reducing ferroptosis by decreasing m^6^A levels and TFRC expression, highlighting a potential therapeutic target for mitigating hemorrhage‐induced cellular damage.^[^ ^157^ ^]^ In addition, upon HBV infection, YTHDF2 undergoes O‐GlcNAcylation at serine 263 via OGT, enhancing its stability and oncogenic functions by reducing ubiquitination. This modification boosts the stability of *MCM2* and *MCM5* mRNAs, facilitating cell cycle progression and tumorigenesis in HBV‐related HCC.^[^ ^158^ ^]^ Moreover, at the onset of type 1 diabetes (T1D), METTL3 in pancreatic β‐cells is upregulated, enhancing m^6^A methylation of key immune mediators and controlling the antiviral response. This response is fine‐tuned by the PTM of METTL3 through S‐nitrosylation at specific cysteines, which influences the stability of oligoadenylate synthase mRNA. Elevated METTL3 levels, sustained via gene therapy, effectively delay T1D progression in mice, highlighting a potential therapeutic strategy targeting METTL3 dynamics (**Table** **3**).^[^ ^159^ ^]^

**Table 3:** Other PTM of RNA modifying proteins and their biological implications.

Protein	Modification	Type	Site	Regulator	Mechanism	Biological Implications	Refs.
METTL14	Methylation	Writer	R255	PRMT1	Enhancing the global mRNA m^6^A levels	Promoting ESC differentiation	[80]
METTL14	Methylation	Writer	IDR	PRMT1	Influencing on RNA methylation activity and m^6^A deposition	Promoting stem cell maintenance and DNA repair	[81]
WTAP	Methylation	Writer	∖	PRMT1	Regulation of m^6^A methyltransferase activity affects NDUFS6 expression	PRMT1 silencing inhibits multiple myeloma cell tumorigenesis	[50]
METTL14	Methylation	Writer	Arginine C‐terminus	PRMT1	Enhancing the ability to bind RNA and catalyze m^6^A modification of mRNAs	Enhancing cell proliferation	[82]
hnRNPQ3	Methylation	Reader	C‐terminal RGG/RXR box	PRMT1	Affecting RNA splicing and degradation	Cellular localization	[83]
hnRNPH1	Methylation	Reader	R233	PRMT1	Reduced binding to mRNA	Preventing alcohol‐induced liver injury	[84]
hnRNPA1	Methylation	Reader	∖	PRMT7 PRMT4 PRMT5	Affecting RNA binding and selective splicing functions	Associating with aberrant splicing and enhanced cell growth	[85]
METTL3	Acetylation	Writer	K263 K388	ACAT1	Blocking ubiquitin‐proteasome mediated degradation	Inhibiting TNBC cell migration and invasion	[113]
METTL3	Acetylation	Writer	∖	IL‐6	Enhancing m^6^A methylation	Promoting breast cancer metastasis	[115]
METTL3	Acetylation	Writer	∖	Sulfatide	Regulating *MTF1* mRNA stability	Influencing HCC progression	[114]
METTL14	Acetylation	Writer	K398	KAT2B	Enhancing METTL14's m^6^A methylation of Spi2a and inhibiting the NF‐κB pathway	Regulating sepsis progression	[115]
ALKBH5	Acetylation	Eraser	K235	KAT8	Promoting demethylation of m^6^A	Contributing to tumorigenesis	[116]
IGF2BP2	Acetylation	Reader	∖	SIRT1	Enhancing transcriptional degradation and impairing v‐ATPase complex expression and lysosomal activity	Promoting tumor survival and aggressiveness	[117]
METTL3	SUMOylation	Writer	K177/211/212/215	SUMO1	Enhancing *SNAI1* mRNA stability	Increasing HCC metastasis	[118]
METTL3	SUMOylation	Writer	K459	SUMO1	Reducing m^6^A levels in mRNAs	Influencing CRC progression	[119]
METTL3	SUMOylation	Writer	∖	RdRp	Affecting its role in viral RNA m^6^A modification	Affecting SARS‐CoV‐2 replication	[120]
NSUN2	SUMOylation	Writer	236–240 amino acids	SUMO‐2/3	Improving stability and nuclear localization of NSUN2	Promoting GC progression	[121]
ALKBH5	SUMOylation	Eraser	∖	ERK/JNK signaling	Enhancing gene expression for DNA repair	Maintaining genomic integrity	[122]
ALKBH5	SUMOylation	Eraser	∖	1‐NP	Enhancing m^6^A modification of FBXW7 and ubiquitin ligase activity on TRF2	Promoting TRF2 degradation and lung fibrosis	[123]
FTO	SUMOylation	Eraser	K216	SIRT1	Reducing mRNA expression	Promoting HCC progression	[124]
IGF2BP2	SUMOylation	Readers	K497, K505, K509	SUMO1	Inhibiting protein degradation via the ubiquitin‐proteasome pathway	Facilitating VM formation	[126]
YTHDF2	SUMOylation	Readers	K571	SUMO1	Enhancing affinity for m^6^A‐modified mRNAs	Promoting cancer progression	[127]
YTHDF2 YTHDF1 YTHDF3	SUMOylation	Readers	K571/K281/K572/K277/K282	PIAS1	Reducing YTHDF2's binding to EBV transcripts	Enhancing resistance to EBV	[128]

## 5 Challenges and Prospects

Investigating PTMs on RNA‐modifying proteins is essential for understanding disease mechanisms and refining targeted therapies. These modifications critically influence RNA splicing, transport, localization, and degradation, all of which are pivotal for gene expression in both normal and diseased states. Elucidating the specific impacts of PTMs on RNA‐modifying proteins provides valuable insights into disease pathogenesis at the molecular level, advancing precision medicine tailored to individual disease profiles. However, the complexity of these regulatory mechanisms poses significant challenges in this field.

Firstly, the exploration of PTMs on RNA modifying proteins is crucial, yet current research in this field is notably sparse. Although PTMs such as phosphorylation, methylation, and ubiquitination are recognized for their roles in regulating these enzymes, the full spectrum of PTMs involved remains largely uncharted. To date, only a limited number of PTMs have been identified as directly impacting RNA modifying proteins. This narrow scope of identified modifications underscores a significant gap in our comprehensive understanding of PTMs functions and their effects on RNA biology. For example, the role of neddylation or palmitoylation in RNA modifying proteins could be crucial but remains unexplored. This oversight could be overlooking critical insights into how cells regulate gene expression in health and disease.^[^ ^160^ ^]^

Secondly, targeting RNA modifying proteins through their PTM has proven to be an effective therapeutic strategy. This approach often involves inducing proteolysis to degrade these proteins, thereby inhibiting their function and impacting cancer progression. For instance, proteolysis targeting chimeras (PROTACs) were engineered using the crystal structure of the METTL3‐14 complex bound to the selective inhibitor UZH2.^[^ ^161^ ^]^ Optimization of these PROTACs involved alkyl linkers that facilitated cell penetration. Validation with FRET‐based biochemical and ubiquitination assays confirmed their efficacy. Specific PROTACs induced significant degradation of METTL3 and METTL14 across multiple cancer cell lines, demonstrating a new approach to modulate epitranscriptomic proteins in cancer therapy. Similarly, WD6305, a potent and selective PROTAC, effectively degrades the METTL3‐METTL14 complex, suppressing m^6^A methylation and enhancing apoptosis in AML cells beyond its parent inhibitor's capacity.^[^ ^162^ ^]^ However, while promising, their clinical application faces significant challenges. Key issues include off‐target effects and specificity concerns due to the widespread expression of E3 ligases, which can lead to unintended protein degradation in normal tissues. In addition, the structural complexity of PROTACs can impact cellular permeability, bioavailability, and stability, potentially limiting their therapeutic efficacy. The “hook effect,” where excessive PROTAC concentrations saturate E3 ligases and reduce degradation efficiency, further complicates their use. Moreover, extensive clinical validation is required to assess safety, efficacy, and optimal dosing in humans, with particular attention to potential toxicity in non‐cancerous tissues. Given the widespread expression of the targeted proteins, it is crucial to ensure that PROTACs exhibit a high degree of specificity to avoid off‐target effects that could compromise the safety of healthy cells. Addressing these concerns will be critical in determining the viability of PROTACs as safe and effective treatments in a clinical setting.

Thirdly, redundancy and compensatory mechanisms within PTM networks represent a sophisticated layer of regulatory control that is pivotal for maintaining cellular function and resilience. The redundancy in PTM networks is manifested by the ability of multiple enzymes to catalyze the same modification on a particular substrate, or on different substrates that converge on similar functional outcomes. For instance, in RNA‐modifying proteins, a variety of kinases can target identical phosphorylation sites, enabling the preservation of RNA processing functions even when specific kinases are inhibited. This enzymatic overlap ensures that critical cellular processes are buffered against single points of failure, thereby enhancing cellular robustness. Moreover, the compensatory mechanisms within PTM networks allow cells to adapt dynamically to internal and external stresses. When one PTM pathway is perturbed, alternative pathways can be upregulated or activated to compensate, ensuring continuity in essential cellular functions. For example, H3R17me2a levels remain unchanged in CARM1 knockout mice due to PRMT6 compensating by methylating this site in vitro. Dual knockout of CARM1 and PRMT6 results in smaller embryos and cells, impaired proliferation, and increased DNA damage, highlighting the functional redundancy of these arginine methyltransferases at the H3R17 site.^[^ ^163^ ^]^ However, this inherent redundancy and compensation also pose significant challenges for therapeutic intervention. The overlapping functions and interconnectivity of PTM networks can obscure the identification of precise molecular targets, as the inhibition of one pathway may lead to the activation of compensatory mechanisms that mitigate the intended therapeutic effects. Furthermore, the specificity of therapeutic agents becomes critical in this context, as unintended modulation of compensatory pathways can lead to off‐target effects or resistance mechanisms that compromise treatment efficacy. Understanding the intricate interplay within PTM networks is therefore essential for the development of targeted therapies that can effectively disrupt pathogenic processes without triggering deleterious compensatory responses. This requires a comprehensive mapping of the PTM landscape, coupled with advanced strategies to selectively modulate specific nodes within these networks. By dissecting the redundancy and compensation within PTM networks, it is possible to identify novel therapeutic targets that exploit these mechanisms for more precise and effective disease interventions.

Fourthly, the study of PTMs on RNA‐modifying proteins, while advancing our understanding of RNA biology, is challenged by the dynamic and spatiotemporal specificity of these modifications, as well as limitations in current detection methodologies. PTMs are highly dynamic, varying with cellular states, tissue types, and physiological conditions. Traditional mass spectrometry (MS), though providing high sensitivity and resolution, often falls short in capturing the real‐time dynamics of PTMs or in measuring the PTM status of a protein across different cellular compartments within the same experiment.^[^ ^164^ ^]^ While emerging technologies, such as high‐throughput ion mobility‐MS, have begun to address the need for observing dynamic changes, such as glycosylation, there remains a pressing need for more precise techniques to fully elucidate the complexity and temporal nature of PTMs. In addition, the reliance on specific antibodies for PTM detection introduces another layer of challenge. Developing highly specific antibodies for particular PTM sites is not always feasible, and cross‐reactivity or the inability to effectively recognize low‐abundance modifications can significantly hinder accurate detection. These limitations underscore the necessity for advancing both the technologies and methodologies available to study PTMs. Overcoming these challenges is crucial for achieving a deeper and more accurate understanding of how PTMs regulate RNA‐modifying enzymes and, by extension, RNA biology as a whole.

Finally, identifying mRNA and noncoding RNA modifications is increasingly recognized as critical to understanding the pathology of human diseases. Aberrant RNA modifications, particularly altered methylation patterns, have been implicated in driving a range of pathological conditions, such as inflammatory disorders, cancer, and metabolic diseases (**Table** **4**). For instance, FTO promotes synovial inflammation and joint damage in rheumatoid arthritis by stabilizing *ADAMTS15* mRNA, while its inhibition reduces arthritis severity.^[^ ^165^ ^]^ Similarly, KHSRP stabilizes FAK pathway mRNAs, facilitating pancreatic cancer progression, with targeting KHSRP suppressing tumor growth.^[^ ^166^ ^]^ Therefore, the precise identification and characterization of these RNA modifications offer significant potential for the development of novel diagnostic tools and targeted therapies. Clinically, such advances could enable the early detection of disease, facilitate monitoring of disease progression, and support the customization of therapeutic strategies based on an individual's unique RNA modification profile. Moreover, targeting specific RNA modifications with small molecules to modulate the activity of RNA‐modifying proteins presents new therapeutic opportunities for managing diseases that remain challenging to treat. As the landscape of RNA modifications becomes increasingly elucidated, their clinical integration into clinical practice could lead to the development of more precise and effective therapeutic strategies.

**Table 4:** Roles and regulatory mechanisms of RNA modification proteins in disease pathogenesis.

Modification	Proteins	Type	Target	Mechanism	Disease	Refs.
m^6^A	METTL3	Writer	SLC7A11	Resistance to ferroptosis	Hepatocellular carcinoma	[20]
			LINC00520	Suppressing ubiquitination of ENO1	Osteosarcoma	[167]
			TRAF1	Increasing *TRAF1* translation and TRAF1 protein	Vascular diseases	[168]
			PTEN	Inhibiting alveolar epithelial cell viability	Acute lung injury	[169]
			RNF43	Regulating oxidative phosphorylation via NDUFS1	Endometriosis	[170]
	METTL14	Writer	GPX4	Promoting ferroptosis and ECM degradation	Osteoarthritis	[171]
			TGFB2	Triggering lipid metabolism reprogramming	PDAC	[172]
			KCTD21‐AS1	Regulating macrophage phagocytosis	Non‐small cell lung cancer	[173]
	WTAP	Writer	DKK3	Promoting DKK3 expression	Diabetic nephropathy	[174]
			TRPML1	Activating Ca^2+^/TFEB signaling pathway	Epilepsy	[175]
	VIRMA	Writer	RASD1	Destabilizing *RASD1* mRNA	Gastric cancer	[19]
	YTHDF1	Reader	MAGED1	Influencing inflammation, glycolysis, ECM‐receptor interaction and PDGF signal pathway	Pulmonary hypertension	[176]
			TINAGL1	Enhancing translation of *TINAGL1*	Esophageal cancer	[177]
	YTHDC1	Reader	CDK6	Repressing CDK6 expression	Retinal vascular injury	[178]
	ALKBH5	Eraser	CARMN	Inhibiting mutant p53‐driven tumor progression	Colorectal cancer	[179]
	FTO	Eraser	ADAMTS15	Regulating synovial aggression and inflammation	Rheumatoid arthritis	[165]
	IGF2BP1	Eraser	RPL36	Attenuating *RPL36* stability and cell proliferation	Benzene hepatotoxicity	[180]
m^5^C	NSUN2	Writer	FABP5	Promoting fatty acid metabolism	Osteosarcoma	[35]
	NSUN6	Writer	∖	Promoting cell cycle progression and proliferation	Colon adenocarcinoma	[181]
	TET1	Eraser	∖	Promoting transcription‐coupled	Cancer	[36]
	YBX1	Reader	PNI‐associated mRNA	Driving neural remodeling	Perineural invasion	[182]
			NS5A	Enhancing HCV RNA stability	Hepatitis C virus	[183]
m^1^A	TRMT10C	Writer	ND5	Leading to mitochondrial dysfunction	Alzheimer's disease	[184]
	TRMT6	Writer	tRNA	Fine‐tuning mTORC1 signaling levels	HSC maintenance	[185]
	TRMT61A	Writer	tRNA	Downregulating MYC/PD‐L1 signaling	HNSCC	[186]
	ALKBH3	Eraser	ATP5D	Regulating cancer cell glycolysis	Cancer	[39]
	YTHDF3	Reader	∖	Modulating macrophage polarization	Abdominal aortic aneurysm	[187]
ac^4^C	NAT10	Writer	COL5A1	Promoting HSP90AA1 expression and metastasis	Gastric cancer	[188]
			SIMALR	Accelerating translation of *ITGB4*/*ITGA6* Promoting the malignant phenotype of NPC cells	Nasopharyngeal carcinoma	[189]
			CEP170	Accelerating tumor proliferation	Multiple myeloma	[190]
m^7^G	METTL1	Writer	tRNA	Enabling efficient mRNA translation	premature senescence	[43]
			FOXM1	Inhibiting PTPN13 expression	Lung adenocarcinoma	[191]
			tRNA	Promoting *GADD45A*/*RB1* mRNAs translation	Breast cancer	[192]
			EGFR EFEMP1	Affecting translation of *EGFR* and *EFEMP1*	Bladder cancer	[193]
	WDR4	Writer	mRNAs	Stabilizing METTL1's structure Enhancing its catalytic activity	Hepatocellular carcinoma	[4]
Ψ	PUS1, PUS3	Writer	tRNA	∖	Intellectual disabilities	[194]
	TruB/PUS4/Cbf5	Writer	tRNA	Impacting mitochondrial translation and biogenesis	∖	[195]
	PUS7	Writer	ALKBH3	Enhancing the translation efficiency of *ALKBH3*	Gastric cancer	[196]

## 6 Conclusion

Collectively, PTMs of RNA‐modifying proteins are crucial for regulating cellular functions and influencing disease progression. PTMs, such as phosphorylation, methylation, acetylation, and ubiquitination, significantly alter the activities, locations, and interactions of proteins involved in RNA modification, including “writers,” “erasers,” and “readers.” These modifications are essential for controlling RNA metabolism and impact gene expression and the cellular response to environmental changes. PTMs are also integral to the development of various diseases, including cancers and neurodegenerative disorders, highlighting their potential as targets for precision medicine. Understanding these modifications is key to unlocking new therapeutic approaches and improving disease treatment strategies.

## Conflict of Interest

The authors declare no conflict of interest.

## Author Contributions

Y.L. and P.L. contributed equally to this work. L.C. and X.Z. conceptualized the idea for the review. L.C., Y.F.L., and P.L. prepared initial drafts of the manuscript. L.C., X.Z., Y.F.L., and L.P. contributed to the writing, graph creation, and manuscript improvement. Y.F.L., J.Z., P.L., Y.L., Z.Y.C., and X.Y.H. contributed to the creation of graphs and tables. All authors reviewed the manuscript and approved the final version.
